# A mechanistic mathematical model of initiation and malignant transformation in sporadic vestibular schwannoma

**DOI:** 10.1038/s41416-022-01955-8

**Published:** 2022-09-12

**Authors:** Chay Paterson, Ivana Bozic, Miriam J. Smith, Xanthe Hoad, D. Gareth R. Evans

**Affiliations:** 1 Division of Evolution, Infection and Genomics, School of Biological Sciences, University of Manchester, Manchester, UK; 2 Department of Applied Mathematics, University of Washington, Seattle, WA USA; 3 Manchester Centre for Genomic Medicine, Manchester University NHS Foundation Trust, Manchester, UK; 4 Radiation Protection Group, Medical Physics, University Hospital Southampton NHS Foundation Trust, Southampton, UK

**Keywords:** CNS cancer, Cancer in the nervous system

## Abstract

**Background:**

A vestibular schwannoma (VS) is a relatively rare, benign tumour of the eighth cranial nerve, often involving alterations to the gene *NF2*. Previous mathematical models of schwannoma incidence have not attempted to account for alterations in specific genes, and could not distinguish between nonsense mutations and loss of heterozygosity (LOH).

**Methods:**

Here, we present a mechanistic approach to modelling initiation and malignant transformation in schwannoma. Each parameter is associated with a specific gene or mechanism operative in Schwann cells, and can be determined by combining incidence data with empirical frequencies of pathogenic variants and LOH.

**Results:**

This results in new estimates for the base-pair mutation rate *u* = 4.48 × 10^−10^ and the rate of LOH = 2.03 × 10^−6^/yr in Schwann cells. In addition to new parameter estimates, we extend the approach to estimate the risk of both spontaneous and radiation-induced malignant transformation.

**Discussion:**

We conclude that radiotherapy is likely to have a negligible excess risk of malignancy for sporadic VS, with a possible exception of rapidly growing tumours.

## Background

Vestibular schwannoma is a benign tumour of Schwann cells on the eighth cranial nerve. It is a relatively rare disease, with a lifetime risk of around 1 in 1000 [1, 2]. The majority of vestibular schwannoma cases carry pathogenic variants on the gene *NF2* and at least one other genetic hit [3]. Age-related risk is described relatively well by a multistage model involving three alterations [4]. This is consistent with a picture in which most cases involve two genetic hits to *NF2*, and one additional genetic insult [3, 5].

In contrast to many other neoplasms, vestibular schwannoma is almost always benign when detected [1, 2]; detailed measurements of tumour growth are possible [6]; and detailed histories of benign disease are often available in cases of malignant transformation [7]. Vestibular schwannomas are very rarely undergo malignant transformation: only around 0.2% of cases develop into malignancy [2, 7]. A recent study on familial neurofibromatosis type II patients (in which pathogenic variants of *NF2* are inherited) suggested an association between radiotherapy and malignant transformation [8]. From such a small sample size, it was impossible to give a quantitative estimate for the excess risk associated with irradiation [9].

Due to the rarity of malignant schwannoma, its pathogenesis is unclear, and the relationships between genomic features, clinical course, and epidemiology are poorly understood [10]. The benign disease is better understood: researchers have been able to use sequencing studies to investigate what genetic alterations have occurred in individual cases [3, 11, 12]. A mathematical model that could connect genomic data to clinical observables would therefore be very useful. Proposed models should pay close attention to specific, plausible mechanisms; as well as summarising the experimental literature in a precise, quantitative form.

One recent example of a mechanistic model connects every parameter to either a population of cells, or the rate of a specific biological process: for example, the mutation rate of individual genes, or the rate of cell division in precursor cells [13]. Several key parameters can be determined directly from the sequences of implicated genes, with no statistical fitting. The main result is an incidence curve for a specific molecular subtype involving alterations to three genes, with the additional advantage that every parameter has a clear interpretation in terms of a specific mechanism [4, 13, 14].

Here, we extend this approach to meet three main goals: first, to describe the incidence of sporadic vestibular schwannoma with a mechanistic model. In this process, we find a new use for existing experimental data in the section “Parameter estimation”, to derive new estimates for biological parameters of interest. Secondly, to use these parameter estimates in a new model for the lifetime risk of malignant transformation. Our work in the section “Modelling sporadic malignant transformation in schwannoma” may help to constrain the genes responsible. Thirdly, to model the excess risk of malignancy following a dose of ionising radiation. At each stage, by relating model parameters to potential observables like variant allele frequencies, we are able draw new inferences from existing data, and suggest informative future experiments.

## Methods

### Modelling incidence of sporadic vestibular schwannoma

The incidence of sporadic vestibular schwannoma is relatively well-described by a three-hit model [4]. Our model is also a three-hit process, but is distinguished from previous efforts by a focus on specific genes; by allowing for hits to occur in any order; and by tying all parameters to underlying mechanisms. For simplicity, we will not study familial or germline *NF2* variants, and focus only on sporadic VS involving somatic *NF2* variants.

**Fig. 1:**
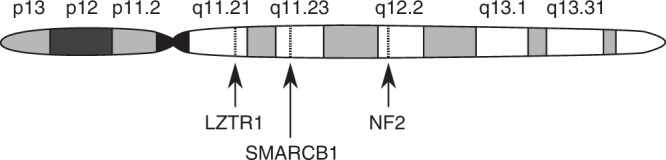
A map of chromosome 22 showing the approximate locations of *NF2* and two other tumour-suppressor genes of possible interest, *SMARCB1* and *LZTR1* [15, 16]. With reference to UCSC Genome Browser [97].

*NF2* displays somatic pathogenic variants in at least 85% of all sporadic vestibular schwannomas according to current estimates, a majority [3, 5]. To reduce the number of unknowns, we consider *only* cases of VS associated with somatic *NF2* loss-of-function. In addition to mutations on *NF2*, which may consist of single-nucleotide variants (SNVs) or indels, loss of heterozygosity (LOH) is also commonly found on chromosome 22 [3, 11]. Loss-of-function of *NF2* can only account for two hits: at least one other gene must be involved. A natural hypothesis is that the third hit is an oncogene (or one of several possible proto-oncogenes). However, the third hit may also be a tumour suppressor on chromosome 22. If both *NF2* and this other tumour suppressor had inactivating mutations on one chromosome, and then LOH removes the other trans allele, both tumour suppressors would then be deactivated by only three events. At least two such tumour suppressors are known, *SMARCB1* and *LZTR1* (see Fig. 1) [15, 16]. We will only consider *SMARCB1* here, and leave the inclusion of *LZTR1* to future work.

We will first define our sporadic incidence model, before showing how to infer its parameters in the section “Parameter estimation”. Our goal will be to derive improved estimates for the underlying parameters from experimental data. These parameters will be critical to our models of malignancy in sections “Modelling sporadic malignant transformation in schwannoma” and “Modelling excess risk of malignancy following irradiation”.

The model has four basic types of parameter: the base population *N*_0_ of progenitor cells from which the tumour derives; the nonsense mutation rates *μ*_*gene*_ for each gene, and the rate *r*_*LOH*_ (22*q*) of LOH on chromosome 22; the selective advantages *s*_*i*_ associated with mutant variants; and patient age *t* [13]. For modelling purposes, a mutation consists of a nonsense mutation caused by either an SNV or an indel. We do not model e.g., frameshift mutations. There is a lack of evidence for an exponential phase in the incidence of benign VS [4]. As a result, we will assume that the three initiating mutations are neutral (*s*_*i*_ ≈ 0) [4, 14]. This reduces the parameters to the initial population *N*_0_, the relevant mutation rates *μ*_*gene*_ and *r*_*LOH*_ (22*q*), and age *t*, the only independent variable [13].

The three alterations in this model consist of two hits to *NF2*, and one hit to either *SMARCB1* or a hypothetical oncogene we will call *GFX*, for “gain-of-function X”. Additional tumour suppressors not on chromosome 22 will be excluded from our discussion of sporadic VS, as this would be a four-event model rather than a three-event model. The model explicitly accounts for the different orders in which the hits can occur. There are three distinct sets of mutations that could result in a schwannoma, and each set may occur in many possible orders:

1. single-copy inactivation of *NF2* → LOH on 22q → gain of *GFX* (3! = 6 different orders)
2. single-copy inactivation of *NF2* → mutation on second *NF2* allele → gain of *GFX* ($\left( 3 \atop 2 \right) = 3$ different orders)
3. single-copy inactivation of *NF2* → LOH on 22q → *SMARCB1* single-copy inactivation (3! = 6 different orders).

Around 65% of the second events in schwannoma tumorigenesis are large chromosomal losses on 22q, overlapping the *NF2* locus. Around 20% are known to be SNVs, with the remainder being unclear [17].

In principle, a complete model should also include hits to *LZTR1*. Due to a lack of available data, we are leaving this to future work.

**Fig. 2:**
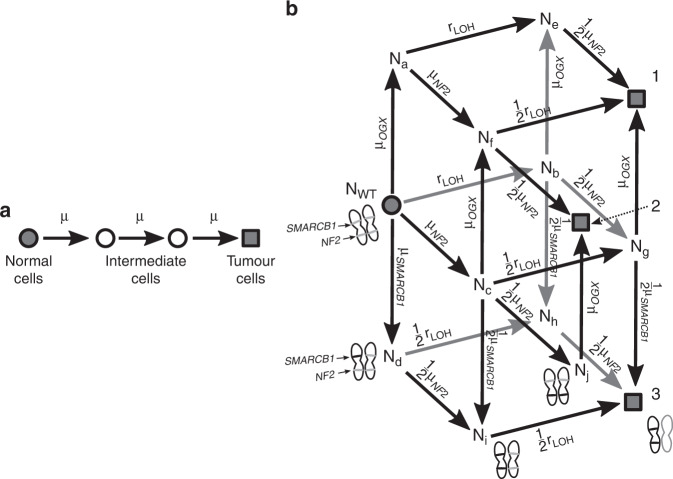
The network structure of two multi-stage models of tumour initiation. **a** A linear multistage model that does not attempt to distinguish between different kinds of alteration [4]. **b** The model in system (1) that allows for all possible orders of occurrence of the three alterations, represented as a directed graph [13, 98]. All the Schwann precursor cells begin on the grey circle, which represents the wild-type population *N*_*WT*_. The intermediate subpopulations are labelled with the corresponding N type from the system (1), and the mutation rates that connect them are labelled with µ_*gene*_ and *r*_*LOH*_. In particular, µ_*GFX*_ represents the SNV/indel rate of a hypothetical oncogene *GFX*. The grey squares 1, 2 and 3 represent neoplastic genotypes with different combinations of genetic alterations.

Each order of events can be represented as a pathway through a network (see Fig. 2): the end state of any of these pathways is a population of tumour cells. These neoplastic mutants are labelled 1, 2 and 3 in Fig. 2. These end states correspond to subtypes of schwannoma with distinct alterations present: LOH will be found in states 1 and 3; pathogenic variants of *SMARCB1* will only be present in state 3; and *NF2* will be doubly mutated in state 2. These different orderings imply the existence of many distinct subpopulations of cells. These intermediate steps, between wild-type progenitor cells and neoplastic tumour cells, are labelled in Fig. 2.

At age zero, the entire population of *N*_0_ progenitor cells are assumed to start in the wild-type state, *N*_*WT*_. Random mutations result in intermediate mutants successively emerging, until a single neoplastic cell results. Rather than treat each subpopulation as a discrete random variable, we will employ a “mean field” or “fluid” approximation, and only track the *mean* subpopulation in each state [13, 14, 18].

Neoplastic cells emerge from the pre-neoplastic cells in a Poisson process. The rate of this process is the rate with which the relevant mutations appear in the subpopulations *N*_*e*_ to *N*_*j*_ (see Fig. 2). The subpopulations *N*_*m*_ labelled in Fig. 2 follow a system of differential equations which can be summarised:

1 $$\frac{{d\vec N}}{{dt}} = M \cdot \vec N$$

where the vector $\vec N = \left( {N_{WT},N_a, \ldots ,N_j} \right)$. The matrix *M* depends on the parameters {*μ*_*NF*2_, *μ*_*SMARCB*1_, *μ*_*GFX*_, r_*LOH*_}, and encodes the allowed transitions in Fig. 2. An explicit form of Eq. (1) is given in Appendix A. The mutation rate *μ*_*gene*_ for a given gene is determined by the error rate *u* per replication per base pair, the cell replication rate *b*, and the number of “sensitive locations” on that gene *n*_*gene*_, [13]:

$$\mu _{gene} = n_{gene}ub$$

and *r*_*LOH*_ is the rate of loss of heterozygosity on chromosome 22. In system (1), the subscript *μ*_*GFX*_ stands for the mutation rate of *GFX*, the hypothetical oncogene. The populations in system (1) have the initial conditions *N*_*WT*_ (*t* = 0) = *N*_0_, *N*_(*othertypes*)_ (*t* = 0) = 0.

The probabilities of developing a schwannoma of subtype 1, 2 or 3 by age *t* follow

2 $$\frac{{dP_1}}{{dt}}	 = \left( {\frac{1}{2}\mu _{NF2}N_e + \frac{1}{2}r_{LOH}N_f + \mu _{GFX}N_g} \right)\left( {1 - P_1} \right) \\ \frac{{dP_2}}{{dt}}	 = \left( {\frac{1}{2}\mu _{NF2}N_f + \mu _{GFX}N_j} \right)\left( {1 - P_2} \right) \\ \frac{{dP_3}}{{dt}}	 = \left({\frac{1}{2}\mu _{SMARCB1}N_g + \frac{1}{2}\mu _{NF2}N_h + \frac{1}{2}r_{LOH}N_i} \right)\left( {1 - P_3} \right)$$

where *P*_*j*_ is the probability for end-node *j* to be reached. These end nodes are labelled in Fig. 2.

Tumours of the vestibular nerve often consist of multiple independent tumour foci on the same nerve, apparently polyclonal, that form a larger mass [19, 20]. This contrasts with models of tumour initiation in other diseases, where each tumour is assumed to be monoclonal [13, 21, 22]. For VS, the emergence of tumours of subtypes 1, 2 or 3 should therefore be treated as *independent* events, and not mutually exclusive events.

After solving systems (1) and (2) for *P*_1_, *P*_2_ and *P*_3_, we can use the independence of these events to find the probability to develop a schwannoma with LOH,

$$Pr\left( {LOH,t} \right) = 1 - \left( {1 - P_1\left( t \right)} \right)\left( {1 - P_3\left( t \right)} \right)$$

the probability to develop a schwannoma in which *SMARCB1* has been inactivated,

$$Pr\left( {SMARCB1^{ - / - }tumour,t} \right) = P_3\left( t \right)$$

and the probability to develop a schwannoma with any of the above alterations,

$$Pr\left( {tumour,t} \right) = 1 - \left( {1 - P_1\left( t \right)} \right)\left( {1 - P_2\left( t \right)} \right)\left( {1 - P_3\left( t \right)} \right)$$

Vestibular schwannoma is a rare disease, so all the final probabilities *P*_*type*_ must be small. When this is the case, we can make the approximation that quadratic and higher order terms (like *P*_1_ *P*_2_ or *P*_1_ *P*_2_ *P*_3_) are zero. The relevant formulae simplify considerably:

$$\begin{array}{*{20}{c}} {Pr\left( {LOH,t} \right) \approx P_1\left( t \right) + P_3\left( t \right)} \\ {Pr\left( {SMARCB1^{ - / - }tumour,t} \right) = P_3\left( t \right)} \\ {Pr\left( {tumour,t} \right) \approx P_1\left( t \right) + P_2\left( t \right) + P_3\left( t \right)} \end{array}$$

System (1) is linear in the intermediate populations *N*_*m*_, and can be readily solved by a variety of methods. Several underlying parameters are presently unknown, so we will first give a symbolic solution, and then determine numerical values of these parameters in section “Parameter estimation”.

The intermediate populations of pre-neoplastic cells must also be small. When this is the case, each probability is approximately a power of age, *P*_*type*_ ≈ *Ct*^*k*^. This can be seen from a power series solution of (1)–(2) around *t* = 0 [13, 14] (see Appendix A). This is analogous to the classic “log-log” behaviour of many cancers [14, 23]. For sporadic vestibular schwannoma, this should be a good approximation at all ages (see Appendix A for a detailed analysis). The solutions for *P*_1_, *P*_2_ and *P*_3_ read

3 $$P_1\left( t \right)	 \approx\; \frac{1}{2}N_0n_{NF2}n_{GFX}r_{LOH}u^{2}b^{2}t^{3} \\ P_2\left( t \right)	 \approx\; \frac{1}{4}N_0n_{NF2}^{2}n_{GFX}u^{3}b^{3}t^{3} \\ P_3\left( t \right)	 \approx\; \frac{1}{4}N_0n_{NF2}n_{SMARCB1}r_{LOH}u^{2}b^{2}t^{3}$$

From Eq. (3), we can find the probability *P*(*tumour,t*) that a tumour has been initiated by age *t*:

4 $$Pr\left( {tumour,t} \right) =	 \; P_{1} + P_{2} + P_{3} \approx \frac{1}{4}N_0n_{NF2}\big( {n_{GFX}\left( {2r_{LOH} + n_{NF2}ub} \right)} \\ 	+ {n_{SMARCB1}r_{LOH}} \big)u^{2}b^{2}t^{3} = At^{3}$$

The constant *A* can be directly related to incidence data and other models, as shown in the section “Parameter estimation” [4]. Also of interest are the probability to develop a tumour in which *SMARCB1* has been inactivated,

5 $$Pr\left( {SMARCB1^{ - / - }tumour,t} \right) = P_3\left( t \right) \approx \frac{1}{4}N_0n_{NF2}n_{SMARCB1}r_{LOH}u^2b^2t^3$$

and the probability to develop a tumour that displays LOH on chromosome 22,

6 $$Pr\left( {LOH,t} \right) = P_1\left( t \right) + P_3\left( t \right) \approx \frac{1}{4}N_0n_{NF2}\left( {2n_{GFX} + n_{SMARCB1}} \right)r_{LOH}u^2b^2t^3$$

**Fig. 3:**
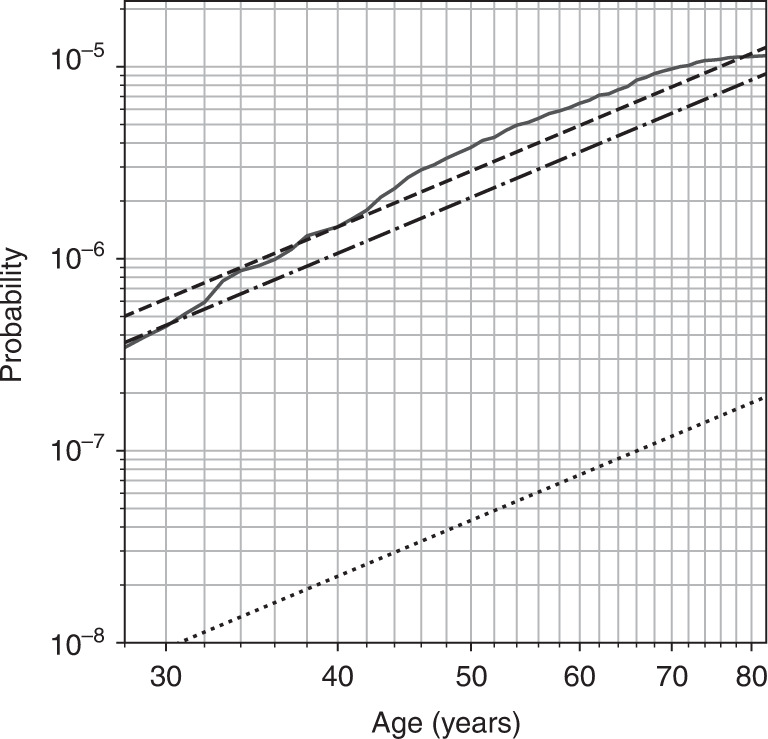
The theoretical probability of developing sporadic vestibular schwannoma (black, dashed curves) and empirical cumulative incidence (solid grey line) as a function of age. The multistage model from Eq. (4) is shown with a black, dashed curve, and has parameters *u* =  4.48 × 10^−10^, the number of sensitive sites on the third hit *n*_*GFX*_ = 2002, and *r*_*LOH*_ = 2.03 × 10^−6^/yr. The dash-dotted curve shows the modelled risk of developing a schwannoma with LOH on 22q from Eq. (6); and the dotted curve shows the risk of developing a schwannoma with a pathogenic variant of *SMARCB1* from Eq. (5). Confidence intervals for the model parameters are established in section “Uncertainties in parameters” and given in Table 1. The empirical curves were derived from incidence data in ref. [1] and corrected for mortality using life tables for the same period from ref. [43].

Equations (5) and (6) are crucial in determining the model parameters in section “Parameter estimation”, as they can be related to empirical frequencies of pathogenic variants. The resulting curves (4), (5) and (6) are plotted in Fig. 3.

#### Parameter estimation

The model in section “Modelling incidence of sporadic vestibular schwannoma” has seven parameters: the initial wild-type population *N*_0_; the cell turnover rate *b*; the mutation rate per base pair *u*; the sensitivity parameters *n*_*NF*2_, *n*_*SMARCB*1_, and *n*_*GFX*_; and the rate of LOH *r*_*LOH*_. These must now be estimated.

#### Precursor cell population and division rate

The base population of precursor cells *N*_0_ can be estimated from anatomical considerations. There are two vestibular nerves, with around 19,000 axons in each [4, 24]. The region on the vestibular nerve from which schwannomas usually originate is ~6 mm long [4], and the approximate spacing between Schwann cells is ~0.5 mm [4]. Thus, the base population in the normal tissue pool *N*_0_ is approximately

7 $$N_0 = 2 \times 19,\!000 \times \frac{6}{{0.5}} = 456,\!000$$

This is higher than R. Woods’ 2003 estimate [4], which we attribute to having a more recent estimate for the number of axons in each vestibular nerve [24].

The rate of cell division *b* in the precursor cell population can be estimated from an experiment on Schwann cell proliferation following injury [25]. Their observed rate of Schwann cell proliferation of 7%/day corresponds to a value of *b* of 25.5/year.

#### Sensitive sites

The effective numbers of sensitive locations *n*_*NF*2_ and *n*_*SMARCB*1_ can be calculated from reference sequences [13, 26, 27]. A site is “sensitive” if a mutation there results in a nonsense codon, truncating the gene. Only sites on the longest open-reading frame are considered. Express *n*_*gene*_ as

8 $$n_{gene} = l_{gene} + m_{gene}$$

where *n*_*gene*_ is the overall number of sensitive sites, *l*_*gene*_ is the number of substitution-sensitive sites, and *m*_*gene*_ is the number of indel-sensitive sites.

To calculate the number of substitution-sensitive sites *l*_*gene*_, count the number of places where a base substitution could result in a nonsense codon. If two possible substitutions can result in a stop codon, count this base twice. Finally, multiply this by 1/3 to account for the fact that only one of the three possible substitutions will result in a nonsense codon. This set of assumptions is equivalent to the Jukes–Cantor model of mutation processes [28].

Several other models of molecular substitution were considered, like Kimura 1980, Tamura and Nei 1993, and Tavaré 1986 [29–32]. In full generality, different rates of transitions/transversions could be represented by redefining

$$\mu _{gene} = \mathop {\sum }\limits_i l_iu_ib + m_{gene}u_{indel}b$$

where *i* runs over the different substitutions, *i* = *C* > *A*, *C* > *T*, *C* > *G* etc., *u*_*i*_ represents the rate of that substitution per replication, *u*_*indel*_ is the mean rate of indels, and *l*_*i*_ represents the number of ways to produce a stop codon with the substitution *i*. Due to a lack of schwannoma-specific data on substitution rates, it was decided not to use more complex models to avoid overfitting, a point elaborated upon in “Discussion”.

Strictly speaking, truncating mutations near the start of the gene might also be expected to be more deleterious than truncating mutations near the end. Something similar is likely to hold for frameshifts. It has also been shown that truncating variants at the very beginning of SMARCB1 lead to reinitiation of translation [33]. The same is thought to be true for NF2, since very early truncating variants cause less severe disease [34]. But to avoid making the model too granular, we have decided to ignore such positional effects, and leave their inclusion to future work.

To calculate *m*_*gene*_, the empirical approximation

9 $$m_{gene} \approx 0.74l_{gene}$$

from Bozic et al. 2010 can be used [35]. However, a more mechanistic way of calculating *m*_*gene*_ is desirable, as this will be an important parameter in the section “Modelling excess risk of malignancy following irradiation”. As a first approximation, we will *only* count sites as sensitive if a deletion there is next to or overlaps a nonsense codon and assume this *always* causes a loss of function. We can now count the number of sites *m*(*k*) where a deletion of length *k* results in a stop codon: TAG, TAA or TGA. Small insertions and deletions have a distribution of possible lengths *k*, with *k* = 1 occurring more often than any other length [36–38].

Calling the length of an indel *k*, with *k* > 0 corresponding to deletions, and *k* < 0 to insertions; denote its frequency of occurrence *f*(*k*). For each possible length *k*, there is a corresponding number of sensitive sites *m*(*k*) on the gene. This is calculated in a similar way for each *k*: for each site on the gene, check if deleting the base pair there, and *k* – 1 bases afterwards, results in a nonsense codon. The number of indel-sensitive sites, *m*_*gene*_, is then the average of *m*(*k*) given the length distribution *f*(*k*):

10 $$m_{gene} = \frac{{\mathop {\sum }\nolimits_{k = - \infty }^\infty m\left( k \right)f\left( k \right)}}{{\mathop {\sum }\nolimits_{k = - \infty }^\infty f\left( k \right)}}$$

In the following, we will assume that insertions (with *k* > 0) are just as likely as deletions (*k* < 0), and *f*(*k*) is symmetric. The *k* < 0 terms will therefore drop out of the sum in Eq. (10).

Given a sequence, we can calculate *m*(*k*) explicitly for all values of *k*. Then, given a length distribution *f*(*k*) such as

$$f\left( k \right) = Aq^{k \vee }$$

*m*_*gene*_ can be computed explicitly given a sequence. We will use *q* = 0.53 for consistency [36], Supplementary Table 5A. Our results were largely insensitive to the exact choice of *q*. Supplemental Python code that computes *l*_*gene*_ and *m*_*gene*_ from EMBL sequences has been provided.

Using reference sequences for *NF2* and *SMARCB1* from the European Nucleotide Archive [26, 27], we find:

$$n_{NF2}	 =\; 251 \times \frac{1}{3} + 44.27 = 135 \\ 	\approx 251 \times \frac{1}{3} \times 1.74 = 142 \\ n_{SMARCB1}	 =\; 143 \times \frac{1}{3} + 37 = 85 \\ 	\approx 143 \times \frac{1}{3} \times 1.74 = 83$$

Which shows that Eq. (9) is a good approximation of the results of Eq. (10) for the genes we consider.

#### Base-pair mutation rates, rates of LOH and *n*_*GFX*_

The number of sensitive sites *n*_*GFX*_ on *GFX* cannot be estimated by a similar counting argument, because the identity of this gene is unknown. Direct measurements of the rate *r*_*LOH*_ of loss of heterozygosity in Schwann cells, and of the per-base mutation rate *u*, are also unavailable.

However, all three parameters can be constrained using measurements of the empirical probabilities *f*_*LOH*_ of LOH on 22q, and *f*_*SMARCB*1_ of pathogenic variants of *SMARCB1*. These can be computed from experimental studies [5, 15, 17, 39]. In a sample size of *n* patients, if *k*_*LOH*_ patients are found to have LOH on 22q, then the relative frequency *f*_*LOH*_ can be estimated as $f_{LOH} = \frac{{k_{LOH} + \frac{1}{2}}}{{n + 1}}$ (see Appendix B for a discussion of additive smoothing). A similar calculation can be done for *f*_*SMARCB*1_, the frequency of pathogenic variants of *SMARCB1*.

The significance of this is that *f*_*LOH*_ and *f*_*SMARCB*1_ are both predicted by our model. From Eqs. (4–6), theoretical predictions for *f*_*LOH*_ and *f*_*SMARCB*1_ can be found:

11 $$f_{LOH}	 = \frac{{Pr\left( {3hitwithLOH} \right)}}{{Pr\left( {3hit} \right)}} = \frac{{P_1 + P_3}}{{P_1 + P_2 + P_3}} \\ 	 \approx \frac{{2n_{GFX}r_{LOH} + n_{SMARCB1}r_{LOH}}}{{2n_{GFX}r_{LOH} + n_{SMARCB1}r_{LOH} + n_{GFX}n_{NF2}ub}}$$

and

12 $$f_{SMARCB1}	 =\, \frac{{Pr\left( {3hitwithSMARCB1} \right)}}{{Pr\left( {3hit} \right)}} = \frac{{P_3}}{{P_1 + P_2 + P_3}} \\ 	 \approx \frac{{n_{SMARCB1}r_{LOH}}}{{2n_{GFX}r_{LOH} + n_{SMARCB1}r_{LOH} + n_{GFX}n_{NF2}ub}}$$

*f*_*LOH*_ and *f*_*SMARCB*1_ are algebraically independent. So, if *f*_*LOH*_ and *f*_*SMARCB*1_ are known experimentally, then two parameters of the model can be fixed. *n*_*GFX*_ and *r*_*LOH*_ can be expressed explicitly in terms of *f*_*LOH*_ and *f*_*SMARCB*1_:

13 $$n_{GFX} = \frac{1}{2}n_{SMARCB1}\frac{{f_{LOH} - f_{SMARCB1}}}{{f_{SMARCB1}}}$$

14 $$r_{LOH} = \frac{{f_{LOH} - f_{SMARCB1}}}{{1 - f_{LOH}}}\frac{1}{2}n_{NF2}ub$$

so that if *f*_*LOH*_ and *f*_*SMARCB*1_ are both known, then the only remaining free parameter is *u*.

A recent study of *NF2* status and LOH in sporadic vestibular schwannoma found that out of 23 patients, 17 had some form of LOH [5]. So, *f*_*LOH*_ = (17 + 0.5)/(23 + 1) = 73%. This includes copy-neutral LOH, by which we understand any reduction in genetic dose at some locus with an otherwise normal karyotype. The primary mechanism of copy-neutral LOH observed in VS is mitotic recombination [11]. Other hypothetical possibilities are trisomy rescue, gain of one chromosome and followed by a loss of or large deletion on the other; and monosomy rescue, a copy number loss followed by subsequent gain [40, 41].

Relevant measurements of *SMARCB1* status were not available, so we performed our own sequencing experiments to determine *f*_*SMARCB*1_. Sequences from 32 sporadic vestibular schwannomas were sourced from the NHS DNA archive in the Manchester Centre for Genomic Medicine, and pathogenic variants of *SMARCB1* were searched for using Sanger sequencing.

Sanger sequencing of *SMARCB1* involved initial amplification of each coding exon, including 50–100 bp flanking intronic sequence per exon, using GoTaq G2 (Promega, Madison, WI, USA). The products were purified using Ampure cleanup beads, and the purified products were used as a template for sequencing PCRs using BigDye Terminator v3.1 (Life Technologies, Paisley, UK). Sequencing PCR products were analysed on an ABI3730xl DNA Analyser (Life Technologies, Paisley, UK). Sequencing chromatograms were aligned to the reference sequence to identify variants.

Our experimental results found no pathogenic variants of *SMARCB1* in a sample of 32 sporadic vestibular schwannomas. The naive estimate of *f*_*SMARCB*1_ is therefore *k*/*n* =  0/32 = zero. This presents a problem, as our estimate for *n*_*GFX*_ diverges when this is the case. As discussed above, we use additive smoothing to regularise *f*_*LOH*_, with a pseudocount of $\alpha = \frac{1}{2}$ (see Appendix B for a further discussion) [42]. Thus,

$$f_{SMARCB1} = \frac{{k + \frac{1}{2}}}{{n + 1}} = \frac{{\frac{1}{2}}}{{32 + 1}} \approx 1.5\%$$

or *f*_*SMARCB*1_ = 1.5%. Together with *n*_*SMARCB*1_ = 85, and *f*_*LOH*_ = 73% imply that

15 $$n_{GFX} = \frac{1}{2} \times 85 \times \frac{{0.73 - 0.015}}{{0.015}} = 2002$$

from (13), and

16 $$r_{LOH} = \frac{{0.73 - 0.015}}{{1 - 0.73}} \times \frac{1}{2} \times 135 \times u \times 25.5 = 4.5 \times 10^3uyear^{ - 1}$$

from (14). The only remaining degree of freedom is now *u*, the error rate per base pair per division. We determine *u* first by fitting a power law,

17 $$Pr\left( {tumour,t} \right) = At^3$$

to incidence data from Evans et al. using non-linear least squares [1]. The coefficient *A* is related to the model parameters by Eq. (4),

18 $$A = \frac{1}{4}N_0n_{NF2}\left( {n_{GFX}\left( {2r_{LOH} + n_{NF2}ub} \right) + n_{SMARCB1}r_{LOH}} \right)u^2b^2$$

The probability to develop a tumour by age *t*, *P*(*tumour,t*), is the cumulative incidence by age *t*, after correcting for survival. Before fitting, the empirical incidence was therefore corrected for mortality using life tables for the period and region of the study [43].

The dataset was also truncated above the age of 80. This is because the relevant population size at ages greater than 80 was very small, and the incidence displays an old-age “plateau” that we suspect is an observational artifact. This amounts to excluding 8 patients from the original dataset of 417, so only 2% of the data has been trimmed [1]. In addition to these modifications to the dataset, our model only accounts for VS associated with somatic *NF2* loss. This occurs in (at least) 85% of cases, so the cumulative incidence data is also rescaled by 85% [3, 5].

Although the results of Carlson et al. suggest that *NF2* may be implicated in 100% of cases, we use AL Håvik et al.’s estimate of 85% as their sample size was larger (*n* = 46 vs. *n* = 23) and both authors’ methods should have a comparable resolution [3, 5].

Equation (17) was fit to the data from Evans et al. using non-linear least squares regression and standard errors obtained from the Hessian matrix [1]. The resulting best-fit value was *A* = 2.26 × 10^−11^/yr^3^, with a standard error of *σ*(*A*) = ±2.95 × 10^−13^. This was not an important source of uncertainty, and was dwarfed by the uncertainty in *n*_*GFX*_ and *r*_*LOH*_ resulting from the small sample sizes of the experiments used to determine *f*_*LOH*_ on 22q and *SMARCB1* alteration frequencies (see the section “Uncertainties in parameters”).

This three-hit model displays a high goodness of fit, with *R*^2^ = 0.989. However, prior work had already established that a three-hit model provided a good fit to this dataset, so replicating this finding with high confidence is not statistically remarkable [4]. The purpose of this procedure was primarily to provide new parameter estimates. These new estimates, in particular *u* and *r*_*LOH*_, will be useful in our study of malignant transformation (section “Modelling sporadic malignant transformation in schwannoma”).

Together with Eq. (18), this value for *A* implies that

$$u = 4.48 \times 10^{ - 10}$$

per base pair per division. This is similar in order of magnitude to the value of *u* ≈10^−9^ estimated by Tomasetti and Bozic, and similar to the range reported by Keogh et al. for brain tissue [44, 45]. This estimate for *u* now allows a concrete numerical value to be placed on *r*_*LOH*_:

19 $$r_{LOH} = 2.03 \times 10^{ - 6} \; yr^{ - 1}$$

This is rather low in comparison to Paterson et al.’ estimate for *r*_*LOH*_ =  1.2 × 10^−4^/yr in colorectal cancer [13]. Possible explanations for this difference are discussed in the section “Sporadic vestibular schwannoma”.

#### Uncertainties in parameters

The sample sizes used to estimate frequencies of *f*_*LOH*_ and *f*_*SMARCB*1_ were relatively small, both with *N* = 20–30 patients. As a result, *n*_*GFX*_, *u*, and *r*_*LOH*_ have important sources of uncertainty that are difficult to quantify with standard methods—i.e., propagation of uncertainty typically assumes normally distributed errors, which is not a valid assumption when sample sizes are small [46].

To address this, distributions for these parameter values were bootstrapped. Randomly resampling regularised datasets for LOH and *SMARCB1* with replacement generates a distribution of estimated values for *n*_*GFX*_, *u*, and *r*_*LOH*_ (see Appendix B) [5, 15, 47]. A 95% confidence interval can then be determined for each parameter from the resulting distribution [48]. These confidence intervals read:

20 $${n_{GFX}} 	 \in {\left[ {315,2442} \right]} \\ u 	 \in {\left[ {3.36 \, \times 10^{ - 10},8.74 \, \times 10^{ - 10}} \right]} \\ {r_{LOH}} 	 \in {\left[ {1.29 \, \times 10^{ - 6},4.93 \, \times 10^{ - 6}} \right] \, yr^{ - 1}}$$

**Fig. 4:**
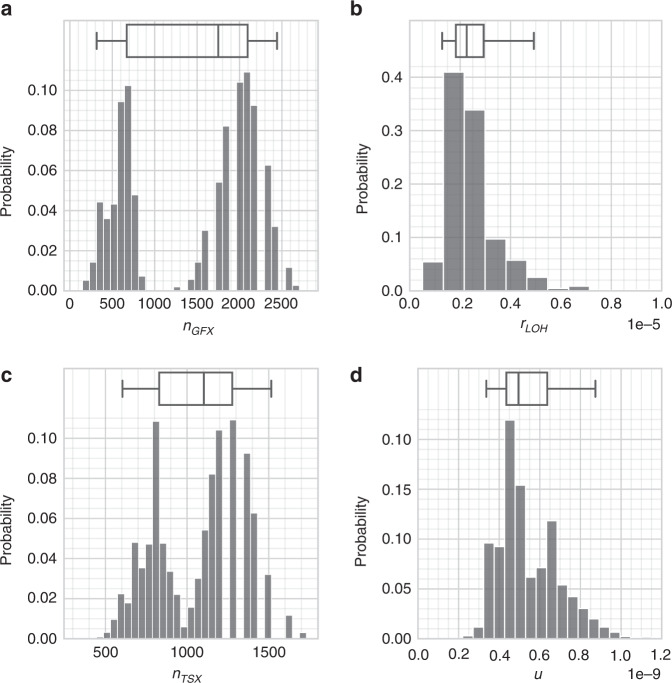
Table of parameter estimates. Bootstrapped distributions for the variables (**a**) *n*_*GFX*_, **b** *r*_*LOH*_, **c** *n*_*TSX*_ (see the section “LOH in malignant schwannoma”), and (**d**) *u*. These were determined by the frequency of LOH from [11], by our own attempted measurements of pathological *SMARCB1* variants, and by incidence data from ref. [1]. Equations (13), (14) and (18) allowed estimates of *n*_*GFX*_, *r*_*LOH*_ and *u* to be generated. The box represents the median estimate and inter-quartile range; the outer whiskers represent the 2.5% and 97.5% quantiles, i.e., the 95% confidence interval [48]. Note that the distributions for *n*_*GFX*_ and *n*_*TSX*_ appear to be bimodal.

In the context of bootstrapped distributions, the “best estimates” from section “Parameter estimation” can be interpreted as modal estimates. Supplemental Python code that implements the bootstrapping procedure has been provided. All parameter estimates and intervals are presented in Table 1 and Fig. 4. The input *f*_*LOH*_ and *f*_*SMARCB*1_ estimates are presented in Table 2.

**Table 1:** Estimates of model parameters.

Symbol	Name	Value	95% CI	Source
*N*_*0*_	Base population	456,000	?	[4, 24]
*b*	Schwann cell division frequency	25.5	?	[25, 90]
*n*_*NF2*_	*NF2* driver locations	135	n/a	This work
*n*_*SMARCB1*_	*SMARCB1* driver locations	85	n/a	This work
*n*_*OGX*_	*OGX* (3rd hit) driver locations	2002	[315, 2442]	This work
*r*_*LOH*_	Rate of LOH on 22q	2.03 × 10^−6^/yr	[1.29,4.93] × 10^−6^ / yr	This work
*u*	Mutation probability	4.48 × 10^−10^	[3.36, 8.74] × 10^−10^	This work
*t*	Age, in years	Variable, 0–100 yrs	n/a	n/a

Summary of estimated parameter values for the sporadic vestibular schwannoma incidence model detailed in section “Parameter estimation”. Uncertainties in the parameters *n*_*GFX*_, *r*_*LOH*_ and *u* were derived by bootstrapping distributions from experimental results (section “Uncertainties in parameters”) [5, 11, 15].

**Table 2:** Frequencies of LOH and pathogenic alleles.

	Vestibular schwannoma	Sporadic schwannomatosis	Spinal schwannoma
f_LOH_	73%*	82%^★^	80%
f_SMARCB1_	1.5%^★★^	8.6%^†^	10%

Estimates of the frequencies *f*_*LOH*_ of LOH and *f*_*SMARCB*1_ of pathogenic mutant SMARCB1 alleles in different studies. ∗: from Carlson et al. [5]; ^★^: from Hadfield et al. [11]; ^†^: from Hadfield et al. [15]; ^★★^: this work. Both spinal schwannoma estimates are from Paganini et al. [16]. All the *f*_*SMARCB*1_ estimates are in the range 0–10%. It may be noted that by the “rule of three” [91–96] that the 95% confidence interval on our *f*_*SMARCB*1_ estimate is [0, 9.4%], suggesting that the differences in *f*_*SMARCB*1_ between the diseases may not be statistically significant.

### Modelling sporadic malignant transformation in schwannoma

Additional mutations may occur as the tumour expands. When the “right” mutations occur together, a cell within the tumour gains a malignant phenotype [49]. In the case of malignant schwannoma, it is not currently clear whether these additional hits are due to oncogenes, tumour suppressors, or a combination of the two. We investigated several possibilities.

It was found during preliminary work that if malignant schwannoma were caused by a single oncogene, then the chances of its emerging from any benign schwannoma would be extremely high, on the order of 100%. A similar conclusion was also reached for any tumour suppressor on chromosome 22. As malignant schwannoma is a rare disease, it is far more likely that malignancy occurs after multiple hits—the simplest hypothesis is that a tumour-suppressor gene that is *not* on chromosome 22 is responsible.

In the following, we call this hypothetical tumour-suppressor gene “*TSX*”. We should bear in mind that there may be *several* such tumour suppressors, presumably associated with distinct molecular subtypes of malignant schwannoma. But for simplicity, we will develop the simplest model that is consistent with experimental observations, and make the operational assumption that there is only one such gene. Models that fully account for genetic diversity and multiple tumour suppressors will be left to future work. If the gene *TSX* needs to be extremely large for the model to make realistic predictions, this would be an early warning sign that we have missed some of this genetic diversity.

Once again, we will adopt a mean-field approach, and model the mean of intermediate populations [13]. Unlike in our approach in the section “Modelling incidence of sporadic vestibular schwannoma”, the underlying “wild-type” population is not constant, but expanding as the tumour grows [14, 50].

We will make the approximation that there is no cell death in the tumour, and all growth occurs at the outermost edge [50]. This is likely to be an underestimate of the real rate of cell division and turnover. But it allows us to eliminate dependence on the time *t* elapsed since tumour initiation, which is not directly observable. This obviates any assumptions about the tumour’s growth curve.

**Fig. 5:**
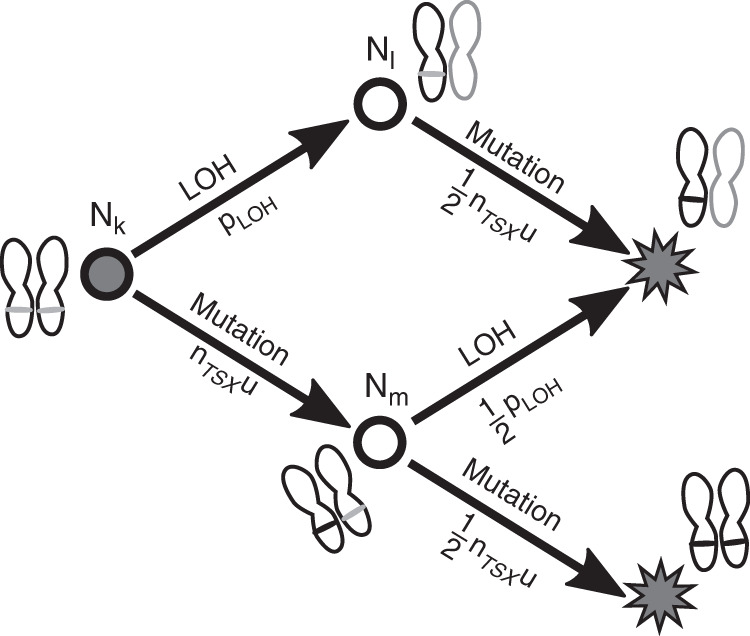
Our 2-hit model of tumour-suppressor inactivation, accounting for all three orders of appearance. The grey circle is the genotype of the initial, benign tumour. The grey stars represent the malignant genotype. The subpopulations from system (21) are labelled *N*_*k*_, *N*_*l*_, *N*_*m*_, and the mutation probabilities indicated below the corresponding arrows.

In each cell division event, there is some probability to create a mutant daughter cell. This can occur through either a mutation (i.e., SNV or indel) on *TSX* with probability *n*_*TSX*_ *u*, or through LOH on the relevant chromosome with probability *p*_*LOH*_. The subpopulations *N*_*k*_, *N*_*l*_, *N*_*m*_ in the expanding tumour (see Fig. 5) obey the following differential equations:

21 $$\frac{{dN_k}}{{dt}}	 =\, g\left( t \right) \\ \frac{{dN_l}}{{dt}}	 =\, n_{TSX}u\frac{{dN_k}}{{dt}} + s_lN_l \\ \frac{{dN_m}}{{dt}}	 =\, p_{LOH}\frac{{dN_k}}{{dt}} + s_mN_m$$

where the growth rate *g*(*t*) of the tumour is some smooth function of time with *g*(0) = 0, and *s*_*l*_ and *s*_*m*_ are fitnesses of the mutant clones. System (21) has the initial conditions: *N*_*k*_ (*t* = 0) = *g*(0) = 0, *N*_*l*_ (*t* = 0) = 0, so that *N*_*k*_ is just

$$N_k = {\int}_{t^{\prime} = 0}^t {g\left( {t{^\prime}} \right)dt{^\prime}}$$

Note that the populations are easier to measure than tumour age *t*.

As in the model from the section “Modelling incidence of sporadic vestibular schwannoma”, we will assume that as soon as a malignant cell emerges in the benign VS tumour, it survives and establishes a growing malignant lineage. The probabilities for a malignant subclone to emerge in a benign VS with and without LOH on the locus of *TSX* follow

22 $$\frac{{dP\left( {malignancywithLOH} \right)}}{{dt}}	 = \left( {\frac{1}{2}n_{TSX}u\frac{{dN_m}}{{dt}} + \frac{1}{2}p_{LOH}\frac{{dN_l}}{{dt}}} \right)\left( {1 - P\left( {malignancywithLOH} \right)} \right) \\ \frac{{dP\left( {malignancywithoutLOH} \right)}}{{dt}}	 = \left( {\frac{1}{2}n_{TSX}u\frac{{dN_l}}{{dt}}} \right)\left( {1 - P\left( {malignancywithoutLOH} \right)} \right)$$

respectively, where *p*_*LOH*_ is the probability for LOH to occur during a single division, and *n*_*TSX*_ is the number of sensitive locations on *TSX*. For the parameters *u* and *p*_*LOH*_, we will use the value of *u* = 4.48 × 10^−10^ from section “Modelling incidence of sporadic vestibular schwannoma”; for *p*_*LOH*_, we will use a value consistent with our previous *r*_*LOH*_ estimate:

23 $$r_{LOH}	 = p_{LOH}b \\ 	 \Rightarrow p_{LOH} = \frac{{r_{LOH}}}{b} = 7.97 \times 10^{ - 8}$$

In the absence of better experimental constraints, we will take *p*_*LOH*_ to be the same for all chromosomes. As *TSX* is currently unidentified, *n*_*TSX*_ cannot be determined a priori from a reference sequence like in section “Parameter estimation”. We constrain *n*_*TSX*_ in the section “Parameter estimation”.

General solutions to system Eq. (21) can be written:

24 $$\begin{array}{*{20}{c}} {N_l = n_{TSX}uN_k + s_le^{s_lt}{\int}_{t{^\prime} = 0}^t {e^{ - s_lt{^\prime}}n_{TSX}uN_k\left( {t{^\prime}} \right)dt{^\prime}} } \\ {N_m = p_{LOH}N_k + s_me^{s_mt}{\int}_{t{^\prime} = 0}^t {e^{ - s_mt{^\prime}}p_{LOH}N_k\left( {t{^\prime}} \right)dt{^\prime}} } \end{array}$$

The fitnesses *s*_*l*_ and *s*_*m*_ relative to the “wild-type” tumour cells *N*_*k*_ are not known. Since both subpopulations *l* and *m* still have one active copy of *TSX*, we will assume these mutations are neutral, *s*_*l*_ = *s*_*m*_ *=* 0. This amounts to assuming that *TSX* is haplosufficient. In this limit, Eq. (24) becomes

25 $$\begin{array}{*{20}{c}} {N_l = n_{TSX}uN_k} \\ {N_m = p_{LOH}N_k} \end{array}$$

Note that *all* explicit dependence on *t* has disappeared, and these depend purely on *N*_*k*_. The risks of different types of malignancy are then given by the solutions of Eq. (22),

26 $$\begin{array}{*{20}{c}} {P\left( {malignancywithLOH} \right)	 = 1 - {{{{{{{\mathrm{exp}}}}}}}}\left( { - n_{TSX}up_{LOH}N_k} \right)} \\ {P\left( {malignancywithoutLOH} \right)	 = 1 - {{{{{{{\mathrm{exp}}}}}}}}\left( { - \frac{1}{2}\left( {n_{TSX}u} \right)^2N_k} \right)} \end{array}$$

The overall risk of malignancy can be calculated from Eq. (26) using the relation

$$1 - P\left( {malignancy} \right) = \left( {1 - P\left( {malignancywithLOH} \right)} \right)\left( {1 - P\left( {malignancywithoutLOH} \right)} \right)$$

from which it follows that

$$P\left( {malignancy} \right) = 1 - {{{{{{{\mathrm{exp}}}}}}}}\left( { - \frac{1}{2}n_{TSX}u\left( {n_{TSX}u + 2p_{LOH}} \right)N_k} \right)$$

We can also note that both *n*_*TSX*_ *u* and *p*_*LOH*_ are small, on the order of 10^−7^. The tumour population *N* and subpopulation *N*_*k*_ must therefore be similar, to within one part in 10^−7^:

$$N = N_k + N_l + N_m = \left( {1 + n_{TSX}u + p_{LOH}} \right)N_k \approx N_k$$

so as a result,

27 $$P\left( {malignancy} \right) \approx 1 - {{{{{{{\mathrm{exp}}}}}}}}\left( { - \frac{1}{2}n_{TSX}u\left( {n_{TSX}u + 2p_{LOH}} \right)N} \right)$$

Finally, we can notice once again that because malignant schwannoma is rare [2], *P* (malignancy) must be very small, *P* ≈ 0.2% ≪ 1, and Eq. (27) simplifies to

28 $$P\left( {malignancy} \right) \approx \frac{1}{2}n_{TSX}u\left( {n_{TSX}u + 2p_{LOH}} \right)N$$

To connect *P*(malignancy) to the observed volume *V* of the tumour, the population *N* must be related to *V*:

29 $$N = \frac{{f_{SC}V}}{{V_{SC}}}$$

where *f*_*SC*_ is the fraction of cells in the tumour that are Schwann cells, *V*_*SC*_ is the volume of an individual Schwann cell (in mm^3^), and *V* the volume of the tumour (also in mm^3^). The majority of cells in schwannomas are not Schwann cells, but macrophages: the best available estimates of *f*_*SC*_ are in the range 0.3–0.5 [51]. Of these, we choose the upper value of *f*_*SC*_ = 0.5. A representative value for the volume *V*_*SC*_ can be set at 1.6 × 10^−6^ mm^3^ (from ref. [52]).

The final expression for the risk of malignancy in terms of tumour volume is thus

30 $$P\left( {malignancy} \right) \approx \frac{1}{2}n_{TSX}u\left( {n_{TSX}u + 2p_{LOH}} \right)\frac{{f_{SC}V}}{{V_{SC}}}$$

**Fig. 6:**
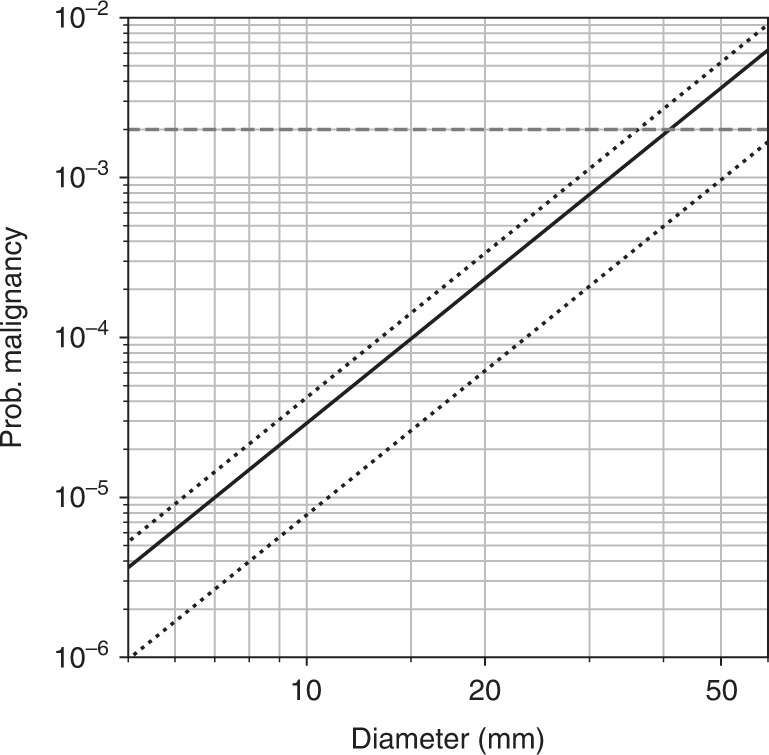
Risk of spontaneous malignant transformation in a benign schwannoma as a function of diameter d in mm, as modelled by Eq. (30). The estimated lifetime risk ≈0.2% is again shown as a horizontal dashed line [2]. The risk curve for our best estimate for *n*_*TSX*_ = 1245 is shown with a solid black line: the upper and lower confidence intervals of 1515 and 604 with dotted lines, above and below (from section “Parameter estimation”).

The tumour volume *V* can be observed, in principle, on MRI scans [53]. Notably, the risk of malignancy is simply proportional to tumour volume *V*. We constrain the number of sensitive sites *n*_*TSX*_ in section “Parameter estimation”, and explore a range of plausible values in Fig. 6.

#### Parameter estimation

The main unknown in this model of malignant transformation is *n*_*TSX*_, the number of sensitive sites on *TSX*. As the identity of this gene is unknown, *n*_*TSX*_ cannot be calculated using a reference sequence. An estimate could help to constrain its identity.

From the SEER study, we know that the lifetime risk of malignant schwannoma ≈0.2% [2]. The model in the section “Modelling sporadic malignant transformation in schwannoma” avoided using “tumour age” as an independent variable, which is usually unobservable: the main result, Eq. (30), is formulated in terms of tumour volume.

If a typical tumour diameter on surgery is 40 mm, the tumour will contain roughly *N* ≈10^10^ cells [54, 55]. If *P*(*malignancy*) ≈0.2% [2], then

31 $$P = \frac{1}{2}n_{TSX}u\left( {n_{TSX}u + 2p_{LOH}} \right)N \approx 0.2\%$$

from Eq. (27). Rearranging (31) for *n*_*TSX*_ is elementary. Given that *u* = 4.48 × 10^−10^, *N* ≈ 10^10^, and *p*_*LOH*_ = 7.97 × 10^−8^ *p*_*LOH*_ = 7.97 × 10^−8^, *n*_*TSX*_ should then be

32 $$n_{TSX} \approx \frac{{p_{LOH}}}{u}\left( {\sqrt {1 + \frac{{2P}}{{p_{LOH}^2N}}} - 1} \right) \approx 1245$$

While this is a high figure, it is not impossible for *TSX* to be one gene: one may compare *n*_*APC*_ = 604 from ref. [13]. It nonetheless seems more likely that several tumour suppressors are involved, and this high estimate for *n*_*TSX*_ indicates a degree of genetic diversity.

This estimate must be qualified by the uncertainties in *u* and *r*_*LOH*_. We can place confidence intervals on *n*_*TSX*_ by a similar bootstrapping procedure to that in the section “Uncertainties in parameters”. Using Eq. (32), one *n*_*TSX*_ estimate can be determined for every pair of *u* and *r*_*LOH*_ values. A distribution for *n*_*TSX*_ can therefore be generated at the same time as those for *u* and *r*_*LOH*_. The resulting 95% confidence interval is

$$n_{TSX} \in \left[ {604,1515} \right]$$

The full bootstrapped distribution for *n*_*TSX*_ is shown in Fig. 4. Supplemental Python code has also been provided.

#### LOH in malignant schwannoma

Another testable prediction is the proportion of malignant tumours that should display LOH. Considered as a subtype of schwannoma associated with a mutation on *TSX*, levels of LOH on chromosome 22 should be similar to that of benign tumours. If malignant transformation involves an additional tumour-suppressor gene, and it is not on chromosome 22, we should also expect to see LOH elsewhere.

This “excess” LOH should be found on the chromosome that carries *TSX*, the hypothetical tumour suppressor from the section “Modelling sporadic malignant transformation in schwannoma”. Studying LOH, especially in the form of copy number changes, has already been widely used to search for tumour suppressors, although it only gives limited information about the precise locus [56]. In this section, we estimate the frequency *f*_*LOH*_ of LOH aggregated across all chromosomes, relate this to *n*_*TSX*_, and estimate a minimum useful sample size for a study.

The parameter *n*_*TSX*_ can be mathematically related to *f*_*LOH*_ by a similar method to that used for *n*_*GFX*_ in the section “Parameter estimation”. This will be useful for two reasons. Firstly, so that future measurements of *f*_*LOH*_ can be used to estimate *n*_*TSX*_ [56]. Secondly, *f*_*LOH*_ can be estimated in advance to judge whether or not such an experiment would be worthwhile.

From Eqs. (25) and (27), it follows that

33 $$f_{LOH}	 = \frac{{P\left( {malignancywithLOH} \right)}}{{P\left( {malignancy} \right)}} \\ 	 = \frac{{1 - {{{{{{{\mathrm{exp}}}}}}}}\left( { - n_{TSX}up_{LOH}N} \right)}}{{1 - {{{{{{{\mathrm{exp}}}}}}}}\left( { - \frac{1}{2}n_{TSX}u\left( {n_{TSX}u + 2p_{LOH}} \right)N} \right)}}	$$

where *f*_*LOH*_ is the excess LOH found at the locus of the unknown tumour suppressor. Again, we know that malignancy is very rare, with a lifetime risk of around 0.2% [2]. In the limit that *P*(*malignancy*) is small, the product *n*_*TSX*_ *up*_*LOH*_ *N* must also be small. Taylor expansion of Eq. (33) with respect to *N* yields

34 $$f_{LOH} \approx \frac{{2p_{LOH}}}{{2p_{LOH} + n_{TSX}u}} + O\left( {n_{TSX}up_{LOH}N} \right)$$

that is, *f*_*LOH*_ should approach a constant value when *N* is sufficiently small. Substituting our estimates of *u*, *p*_*LOH*_ and *n*_*TSX*_ into (28), one can show that this approximation holds when *N* ≪ 10^12^ *cells*. A tumour with 10^12^ cells would be 18 cm in diameter. This is a hundred times more massive than the majority of schwannomas [54, 55]: it should not even be possible for a tumour this large to form inside the cerebellopontine angle. Equation (34) should therefore be a good approximation.

Because *f*_*LOH*_ is insensitive to tumour size *N*, it should be possible to estimate *n*_*TSX*_ from experimental *f*_*LOH*_ figures even when the sizes and ages of the sampled tumours are unknown. Equation (34) implies:

35 $$n_{TSX} \approx \frac{{2p_{LOH}}}{u}\frac{{1 - f_{LOH}}}{{f_{LOH}}}$$

Without an LOH survey of malignant schwannoma samples, this formula cannot yet be used to provide a new estimate of *n*_*TSX*_. It is presented here for future work.

Returning to the second point about whether or not this experiment would be realistic, we should now estimate *f*_*LOH*_. Substituting the estimates *p*_*LOH*_ = 7.97 × 10^−8^, *u* = 4.48 × 10^−10^ and *n*_*TSX*_ ≈1245 into Eq. (34) yields

$$f_{LOH} \approx \frac{{2p_{LOH}}}{{2p_{LOH} + n_{TSX}u}} = 22\%$$

This is a large enough value that a pilot study with only 15 samples is likely to detect something: the probability that *no* samples display excess LOH, assuming the above estimates, is (1–0.22)^15^ = 2.4%.

It is notable that (34) is independent of tumour size *N*. A study of biopsies from malignant VS tumours should therefore not detect an association between LOH and tumour size. It should be remembered that this assumes selective neutrality of *s*_*l*_ and *s*_*m*_—i.e., *TSX* is haplosufficient.

### Modelling excess risk of malignancy following irradiation

The main mechanism of mutagenesis following radiation is the induction and misrepair of double-strand breaks (DSBs) [57–60]. Our model of radiation mutagenesis can be summarised in simple terms: following a large dose of radiation *D*, a DSB occurs at a given base pair with some small probability *p*_*DSB*_ (*D*). The DSB is then repaired, with a probability *ϵ* of faulty repair. There are *m*_*TSX*_ such sites on gene *TSX* where misrepair results in *TSX* being deactivated. If a DSB occurs on a gene, *and* the repair was faulty, *and* it was on one of the sensitive sites, then one copy of *TSX* will be inactivated. These are taken to be independent events. The probability *P*(*TSXlost*) to inactivate one copy of *TSX* in a given cell is therefore

36 $$P\left( {TSXlost} \right) = p_{DSB}\left( D \right) \times P\left( {misrepair} \right) \times \left( {number\;of\;sensitivesites} \right) = p_{DSB}\left( D \right){\it{\epsilon }}m_{TSX}$$

The repair error rate *ϵ*, probability of DSB induction *p*_*DSB*_, and number of radiosensitive sites *m*_*TSX*_ now need to be specified.

There are two main mechanisms of repair following irradiation and the induction of double-strand breaks. These are non-homologous end joining (NHEJ), and homologous repair (HR) [58–60]. NHEJ is thought to have a high error rate, and HR a low (but nonzero) one [58]. In addition to NHEJ, there are several other more mutagenic repair mechanisms that take over when HR is suppressed [57]. At low-dose rates, HR is supposed to be the primary repair mechanism, with NHEJ and alternative mechanisms taking over at higher dose rates [57, 58, 61]. Misrepair is much more common at large dose rates of radiation, in excess of 0.5 cGy/min [61–63].

DSB misrepair is associated with the introduction of small insertions and deletions (“indels”) [58]. To model the number of sensitive sites, we will use the parameter *m*_*TSX*_ from the section “Modelling incidence of sporadic vestibular schwannoma”, because this was the number of indel-sensitive locations. From Eq. (9), *m*_*TSX*_ ≈ 0.74*l*_*TSX*_, or in terms of *n*_*TSX*_, the total number of sensitive sites on *TSX*,

37 $$m_{TSX} = \frac{{0.74}}{{1.74}}n_{TSX} = 0.42n_{TSX}$$

The therapies we are modelling have high dose rates, on the order of several Grays per minute: this is far outside the low-dose rate regime studied by Stenerlöw et al. [63]. The repair in this regime will therefore be relatively error-prone, similar to that studied by Rothkamm et al. In this high dose rate regime, as many as 50% of the repairs may be faulty [62]. Strictly speaking, the error rate should depend on both dose *D* and dose rate $\dot D$: i.e., ${\it{\epsilon }}\left( {D,\dot D} \right)$. In the absence of sufficiently well-developed models, we will instead take *ϵ* = 50% = constant [62], and leave more detailed models of DSB repair to future work. The figure of *ϵ* = 50% is a pessimistic upper bound: this section should then establish an upper limit on the associated risk.

The only term in the above model that has not been fixed is *p*_*DSB*_ (*D*), the probability for a DSB to be induced. This has been measured directly, at the same energies and doses of gamma rays that are used in therapy [64]. In the 0–50 Gray regime, the results are well-described by a simple linear interpolation,

38 $$p_{DSB}\left( D \right) \approx kD$$

with *k* = 3.90 × 10^−7^ per Gray per base pair. Over the entire genome, this corresponds to about 300 induced DSBs per Gray of radiation. This is substantially higher than older measurements of mutagenesis using X-rays—this is consistent with the understanding that gamma radiation is more mutagenic [61, 63, 64].

To result in malignancy, the cell carrying the new mutation has to survive. Following a dose of ionising radiation, a fraction of any population of cells will fail to replicate, and die. We will assume that all cells in the tumour have the same probability *S*(*D*) to survive the dose *D* administered. Following current models in radiobiology, we will use a “linear-quadratic” form for *S*(*D*) [65, 66]:

39 $$S\left( D \right) = {{{{{{{\mathrm{exp}}}}}}}}\left( { - \alpha D - \beta D^2} \right)$$

and take *α* = 0.77 Gy^−1^ and *β* = 0.31 Gy^−2^ [65, 66].

Combining Eqs. (36), (38), and (39), the probability that after a dose of radiation, a given cell loses one copy of *TSX and survives* is

40 $$P\left( {TSXlostandcellsurvives} \right) = kD{\it{\epsilon }}m_{TSX}S\left( D \right)$$

Because *kD* will be on the order of 10^−5^, and *ϵ* and *S* both on the order of 1, we can only expect this probability to be very large when *m*_*TSX*_ ≈10^5^. The largest known gene in the human genome, dystrophin, is 2.5 Mb long—the majority are much smaller [67]. It should be safe to assume that any candidate *TSX* will be short enough that the probability Eq. (40) is much smaller than 1.

Not all cells will become cancerous when hit by a dose of radiation. Cells that still have two copies of *TSX* can lose one copy and still retain one functional copy. Cells that have already lost one copy of *TSX* may become malignant if they lose the other. The only subpopulations that will be sensitive to irradiation are therefore *N*_*l*_ and *N*_*m*_ from Fig. 5.

The probability that a tumour gains a new malignant clone following irradiation should therefore be:

41 $$P\left( {TSXlostinatleast1cell} \right)	 = 1 - \left( {1 - kD{\it{\epsilon }}m_{TSX}S\left( D \right)} \right)^{N_l + N_m} \\ 	 \approx 1 - {{{{{{{\mathrm{exp}}}}}}}}\left( { - k{\it{\epsilon }}m_{TSX}DS\left( D \right)\left( {N_l + N_m} \right)} \right) \\ 	 \approx 1 - {{{{{{{\mathrm{exp}}}}}}}}\left( { - k{\it{\epsilon }}m_{TSX}\left( {n_{TSX}u + p_{LOH}} \right)DS\left( D \right)N_k} \right) \\ 	 \approx 1 - {{{{{{{\mathrm{exp}}}}}}}}\left( { - k{\it{\epsilon }}m_{TSX}\left( {n_{TSX}u + p_{LOH}} \right)DS\left( D \right)N} \right)$$

where we have used the solutions (25) and the approximation *N*_*k*_ ≈ *N*. The total number of cells in the tumour *N* can again be related to the volume of the tumour using Eq. (29).

Equation (41) gives the probability that the gene *TSX* will be lost. This can only result in malignancy if it has not been lost already. The probability to have developed a malignancy after a dose *D* should be

42 $$Pr\left( {malignancy} \right) =	\; P\left( {TSXlostinatleast1cell} \right)Pr\left( {nomutantclonebefore} \right) \\ 	+ Pr\left( {mutantclonebefore} \right) \\ =	\; P\left( {TSXlostinatleast1cell} \right)\left( {1 - Pr\left( {mutantclonebefore} \right)} \right) \\ 	+ Pr\left( {mutantclonebefore} \right)$$

Current practice defines the excess risk *E*.*R*. of malignant transformation as the *absolute risk difference* [68]: the difference between the probability *Pr*(*malignancyafterirradiationdoseD*) and the probability of a malignancy having occurred spontaneously beforehand, *Pr*(*mutantclonebefore*):

43 $$E.R.\left( D \right) = Pr\left( {malignancyafterirradiationdoseD} \right) - Pr\left( {mutantclonebefore} \right)$$

From Eqs. (42) and (43), it should be clear that

$$E.R.\left( D \right) = \left( {1 - Pr\left( {mutantclonebefore} \right)} \right)Pr\left( {TSXlostinatleast1cell} \right)$$

and finally, substituting Eq. (27) for *Pr*(*mutantclonebefore*) into the above yields the main result,

44 $$E.R.\left( D \right) = 	\left( {1 - {{{{{{{\mathrm{exp}}}}}}}}\left( { - k{\it{\epsilon }}m_{TSX}\left( {n_{TSX}u + p_{LOH}} \right)DS\left( D \right)N} \right)} \right) \\ 	{ \times {{{{{{{\mathrm{exp}}}}}}}}\left( { - \frac{1}{2}n_{TSX}u\left( {n_{TSX}u + 2p_{LOH}} \right)N} \right)}$$

Some initial observations can be made. E.R.(*D*) initially increases linearly in dose *D*, with no lower threshold. At some point, the exponential fall-off of *S*(*D*) will cause E.R. to peak, then decrease rapidly. The *stochastic* effect of radiation-induced mutations is eventually overcome by the *deterministic* effect of necrotic cell death. It is remarkable that the linear dependence at low doses, below around 2 Gray, is consistent with the linear no threshold model [65].

#### Dose fractionation

Equation (44) assumes that the dose *D* is delivered in a single fraction. However, it is more common to deliver treatments in multiple fractions. This has been demonstrated to provide effective tumour control whilst also reducing side effects in healthy tissue [69, 70]. Fractionation schedules need to be chosen in order to balance what are typically referred to as the “5 R’s of radiotherapy”: repopulation, repair, redistribution, reoxygenation and radiosensitivity. Fractionation is beneficial from the perspective of tumour control, as it allows for the redistribution and reoxygenation of tumour cells during the course of treatment, whilst also allowing healthy cells a chance to repair and repopulate.

Fractionated doses should be administered so that tumour cells have no or little time to recover. Delivered in rapid enough succession, the *total* dose *D* will be what matters, and a tiny proportion of tumour cells will survive due to the rapid fall-off in *S*(*D*). How closely doses need to be spaced to prevent tumour cell recovery likely depends on the cell-cycle time. This is the rationale behind scheduling fractions to be delivered every day, or every 2 days.

A typical cell-cycle time for Schwann precursor cells is not known to a high degree of precision. The *b* = 25.5/yr estimated in section “Parameter estimation” corresponds to a cell-cycle time of around 14 days, but this was for wild-type precursor cells, not tumour cells. We can estimate the cell-cycle time for tumour cells using tumour expansion speed *c* and Schwann cell volume *V*_*SC*_ via a scaling argument:

45 $$c	 \approx V_{SC}^{1/3}b \\ \Rightarrow b	 \approx \frac{c}{{V_{SC}^{1/3}}} \\ 	\approx 90 - 272yr^{ - 1}$$

with *V*_*SC*_ = 1.6 × 10^−6^ mm^3^ from ref. [52], as in Eq. (29); and *c* = 1–3 mm/yr from ref. [6]. This rough estimate should be consistent with estimates from both Fisher–Kolmogorov and Eden model approaches to solid tumour growth [71, 72]. Note that this estimate is faster than the precursor cell division rate, which is consistent with tumour cells having a small selective advantage.

The surface growth rate *b* estimated in this way corresponds to a cell division timescale of 1–4 days. As long as fractionated doses are spaced more closely than this, they should have the same effect on tumour cells as a single large dose, and the excess risk will be governed by Eq. (44), and not Eq. (47), below.

However, enough is unknown about the dynamics of Schwann cells in vivo that we should also consider a pessimistic scenario, in which tumour cells manage to fully recover between fractionated doses. This should give us an upper bound—the worst-case scenario—on the risk of properly fractionated therapy, with Eq. (44) giving the lower bound—the best case scenario.

In this worst-case scenario, we treat the risk of mutations being induced following each fractional dose as *completely independent events*:

$$P\left( {TSXlostafter\;Fdoses} \right) = 1 - \left( {1 - P\left( {TSXlost,D/F} \right)} \right)^F$$

which implies

46 $$P\left( {TSXlostafterFdoses} \right)	 = 1 - \left( {1 - P\left( {TSXlost,D/F} \right)} \right)^F \\ 	 = 1 - {{{{{{{\mathrm{exp}}}}}}}}\left( { - k{\it{\epsilon }}m_{TSX}\left( {n_xu + p_{LOH}} \right)N\frac{D}{F}S\left( {D/F} \right)} \right)^F \\ 	 = 1 - {{{{{{{\mathrm{exp}}}}}}}}\left( { - k{\it{\epsilon }}m_{TSX}\left( {n_xu + p_{LOH}} \right)NDS\left( {D/F} \right)} \right)$$

and the excess risk associated with a dose *D* delivered in *F* fractions is

47 $$\begin{array}{*{20}{c}} {E.R.\left( {D,F} \right) = \left( {1 - {{{{{{{\mathrm{exp}}}}}}}}\left( { - k{\it{\epsilon }}m_{TSX}\left( {n_{TSX}u + p_{LOH}} \right)NDS\left( {D/F} \right)} \right)} \right)} \\ { \times {{{{{{{\mathrm{exp}}}}}}}}\left( { - \frac{1}{2}n_{TSX}u\left( {n_{TSX}u + 2p_{LOH}} \right)N} \right)} \end{array}$$

The likelihood of inducing a new mutation is essentially unchanged, but the likelihood of its *survival S*(*D*/*F*) now depends strongly on the number of fractions *F*. This has important implications in section “Excess risk of malignancy following radiotherapy”.

## Results

### Sporadic vestibular schwannoma

The answer to our first research question, on modelling the risk of benign vestibular schwannoma, is that observed incidence can be explained well with a mechanistic, three-hit model involving *NF2*, *SMARCB1*, and a hypothetical oncogene *GFX*. A comparison of the model’s fit to empirical cumulative incidence curves from Evans et al. is shown in Fig. 3 [1]. The simple model in section “Modelling incidence of sporadic vestibular schwannoma” has a clear interpretation for each of its parameters, which allowed us to find new estimates for the mutation rate *u* of SNVs and indels, and the rate *r*_*LOH*_ of LOH on 22q, which improve on current estimates for Schwann cells (see Fig. 1). The technique of estimating parameters and bootstrapping uncertainties from measurements of the relative frequencies *f*_*LOH*_ and *f*_*SMARCB*1_, derived from experimental data, should also be applicable to other neoplasias (see section “Uncertainties in parameters” and Fig. 1).

The estimation technique for *r*_*LOH*_ is very sensitive to the experimental frequency *f*_*LOH*_ of LOH events. If an experiment can’t detect all forms of LOH present, *r*_*LOH*_ will be underestimated. We have tried to anticipate this by drawing on studies that explicitly account for copy-neutral LOH [5]. Nonetheless, it is natural to ask why our estimate of *r*_*LOH*_ is almost 70 times lower than a previous estimate for colorectal cancer [13]. First, the cell division rate *b* is much slower in glia (*b* = 25.5/yr [25]) than in the colonic crypt (*b* = 73/yr [73]), so the values of *r*_*LOH*_ cannot be directly compared. We should instead compare *p*_*LOH*_ = *r*_*LOH*_/*b*, the probability of an LOH event per division, and account for uncertainties in both estimates of *p*_*LOH*_.

**Fig. 7:**
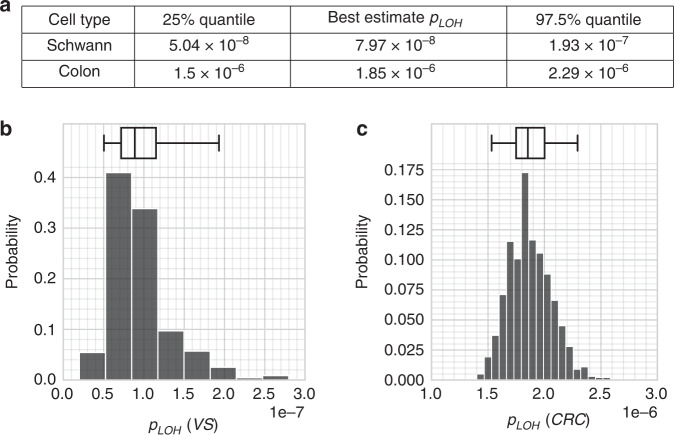
Relative frequency *p*_LOH_ of LOH in Schwann cells and colonic crypt cells. **a** Quantiles for bootstrapped distributions for *p*_*LOH*_ estimates (above). **b** Visualisations of the distributions for Schwann progenitor cells, and (**c**) colorectal crypt progenitor cells, demonstrating a substantial difference in order of magnitude. The estimates for colon cells were derived from Huang et al. [74] and Paterson et al. [13]. The estimates for Schwann cells are original to this work.

Performing a similar bootstrapping procedure for the estimates of *p*_*LOH*_ for colorectal crypt cells by resampling data from Huang et al. and using the same calculation as Paterson et al. to determine *p*_*LOH*_ confirms that there is still a statistically significant difference—a factor of 23 (see Fig. 7) [13, 74]. This stark difference suggests that rates of LOH are simply different between glial cells and colonic crypt cells, even controlling for cell division frequency, just as mutation rates vary substantially between tissues and tumour types [35, 73, 75]. Herrero-Jimenez et al. reported that *p*_*LOH*_ may vary by a factor of 30 between colonic crypt and hematopoietic stem cells, so a difference of a factor of 23 between different tissues is quite possible [76]. Such striking differences in *p*_*LOH*_ and mutation rates with tissue may suggest a coupling of mechanisms of DNA (mis)repair to cell differentiation [77].

As a result of the efforts of the Pan-Cancer Analysis of Whole Genomes Consortium (PCAWGC), a wealth of gene- and tumour-specific data on the prevalence of LOH is now available [75, 78]. While neither study seems to contain vestibular schwannoma, MPNST, or related tumours such as meningioma or oligodendroglioma, Gerstung et al. do report several interesting copy number alterations and hits to *TP53* in glioblastoma multiforme (GBM). Copy number losses on 17p and coding mutations in *TP53* occur with a similar frequency, *f*_*LOH*_ (17*p*) ≈27% ([75] Fig. 3g). As such, our finding of a comparable rate of LOH *r*_*LOH*_ ≈ 2.03 × 10^−6^/yr on 22q and mutation rate *μ*_*NF*2_ ≈1.5 × 10^−6^/yr (see Table 1) of *NF2* might hold more generally for tumours derived from glial precursor cells: while it is not possible to say with certainty without additional modelling, *μ*_*TP*53_ and *r*_*LOH*_ (17*p*) (on the same loci) must at least have similar orders of magnitude for alterations to occur with such similar prevalence. Furthermore, the same study observed copy number losses on 22q in 44% of cases, which is consistent with a role for *NF2* in GBM [79]. Conversely, loss-of-function variants in *TP53* are unheard of in vestibular schwannoma [80].

This same work of the PCAWGC found that these hits to *TP53* must occur “early” rather than “late”, and LOH on 22q must occur “late” in GBM [75]. If there is a role for *NF2* in GBM, it is therefore likely to be a relatively “late” driver. In contrast, our model for malignant transformation in vestibular schwannoma suggests that the last alterations before malignancy should be in an unidentified tumour suppressor (or suppressors) *TSX*, with *NF2* already being inactivated or lost in the benign tumour. If there is any role for *TP53* in malignant schwannoma, it can therefore be expected to be “late”, with *NF2* being “early”. This is the reverse of the case in GBM [75]. Loss-of-function of *TP53* has been speculated to be involved in the malignant transformation of VS [7].

The number of sensitive sites on the third hit *GFX*, *n*_*GFX*_, was found to be 2002. This high value may indicate that there are several oncogenes that contribute to sporadic incidence, representing a high level of genetic diversity. This is also likely to be a slight overestimate of the real figure, and may be revised once *LZTR1* is taken into account.

The posterior distribution for *n*_*GFX*_ was found to be bimodal, with two peaks around 700 and 2000 (see Fig. 4). We believe this is due to the low rate of *SMARCB1*-positive samples in our experiments, which implies a high uncertainty in Eq. (13). A larger sample size for *SMARCB1* levels would be very helpful to better constrain *n*_*GFX*_.

It is also interesting to note that the theory developed in section “Modelling incidence of sporadic vestibular schwannoma” predicts that there should be no association between the relative frequency *f*_*LOH*_ of LOH and patient age. The same holds for *f*_*SMARCB*1_. This is a consequence of neutrality: a change in allele frequency over time would indicate selection for one of the intermediate genotypes in Fig. 2 [81]. Previous work also suggests that indirect knowledge of selective advantages may be contained in the orders of appearance of mutations, even when the full dynamics over time are not available [13].

### Malignant transformation

Our second goal was to establish the risk of sporadic malignant transformation in an untreated schwannoma. The model in the section “Modelling sporadic malignant transformation in schwannoma” culminates in Eq. (30): a simple linear relationship between risk of malignancy and tumour volume *V*. The most interesting finding here is that most of the observed lifetime risk of malignancy can be explained by sporadic incidence (see Fig. 6) [2]. If malignant transformation is associated with the loss-of-function of an unidentified tumour suppressor *TSX*, then plausible values for *n*_*TSX*_ and tumour volume can explain much of the observed lifetime risk (see Fig. 6).

This should be qualified by the uncertainty involved. The tumour suppressor *TSX* is currently unidentified, so a wide range of estimates for risk can be produced by adjusting *n*_*TSX*_. Furthermore, our early estimates of *n*_*TSX*_ suggest a value on the order of 1245, which while not impossible is relatively high. This may suggest that there is a degree of genetic diversity that our modelling approach has not yet captured. On the other hand, there are tumour suppressors implicated in other cancers known to have higher *n*_*gene*_ values, so there is some chance that one gene explains most of the incidence [13].

To try and locate the genes involved in malignancy, we sketch an experimental study in the section “LOH in malignant schwannoma”. Our calculations suggest that “excess” LOH should be a frequent enough occurrence to detect by profiling at least 15 tumours. This is probably achievable by looking for gross copy number changes and SNP arrays—but higher resolution approaches like next-generation sequencing would be much more sensitive to copy-neutral LOH and smaller deletions [82–85]. While not a detailed experimental design, this sketch suggests that such an experiment should be feasible.

As was the case with the model of sporadic incidence, these levels of LOH should not be associated with tumour size in any significant way. This is a consequence of neutrality: an association with tumour size would indicate selection for one of the intermediate mutants [81], and thus point to haploinsufficiency of the implicated gene.

### Excess risk of malignancy following radiotherapy

**Fig. 8:**
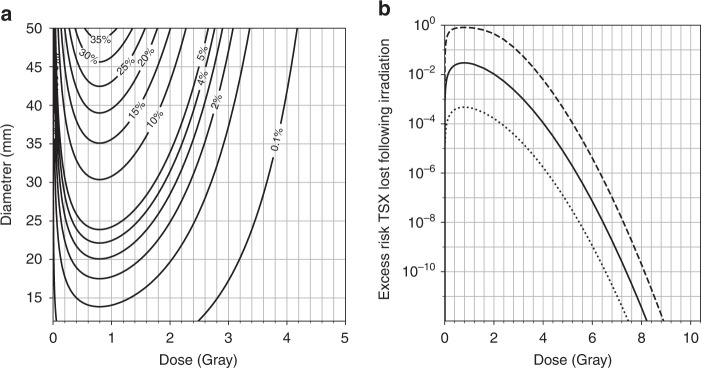
Our model for the excess risk of malignancy associated with irradiation, from Eq. (44). This model is based on the frequency of DSB induction by irradiation, and accounts for both tumour volume and cell survival (see the section “Modelling excess risk of malignancy following irradiation”) [8, 64, 65]. The dose is assumed to be delivered in a single fraction, as in stereotactic radiosurgery. Fractionated radiotherapy should have a similar dose–response relationship if scheduled properly (see the section “Dose fractionation”). **a** The excess risk of a radiation-induced DSB deactivating a tumour suppressor as a function of both tumour diameter (*y* axis) and dose (*x* axis), for *n*_*TSX*_ = 1245 (best estimate from section “Parameter estimation”) and *m*_*TSX*_ = 0.42 × 1245 = 523 (from Eq. (37)). **b** The risk of deactivating a tumour suppressor in a tumour of diameter 20 mm as a function of dose: for example values of *n*_*TSX*_ of 100 (lower curve, dotted), 1245 (middle curve, solid), and 104 (upper curve, dashed), and values of *m*_*TSX*_ = 0.42*n*_*TSX*_.

Our final goal was to develop an a priori model of the excess risk of malignant transformation following irradiation. For both unfractionated radiosurgery and for fractionated radiotherapy under ideal conditions, the excess risk of malignancy was found to be totally negligible for realistic doses. As can be seen in Fig. 8, the excess risk of malignant transformation for a total dose of >10 Gray is remote, less than 1 in a billion. This should hold for a wide range of tumour sizes and values of *n*_*TSX*_. This is consistent with previous studies that found radiotherapy had no detectable association with new malignancies [3, 86].

For lower doses, on the order of 1–4 Gray, the risk is much greater. These doses are much lower than therapeutic targets, so current best practice should have a minimal risk. However, for cases where there are many diffuse tumours in the same region, it may not be possible to target individual tumours with optimal dosing. The relative risk for these cases should be revisited as a priority, ideally combining the above model with medical imaging data.

It must be emphasised that our conclusions about radiotherapy are theoretical, and an indirect extrapolation from experiments and incidence studies. We have tried to account for the inevitable uncertainty in this by erring on the side of pessimism: for example, with regards to the DSB repair error rate *ϵ* in section “Modelling excess risk of malignancy following irradiation”. This is so that we can estimate a reasonable upper bound on risk, despite the unknowns.

If doses are spaced so that fewer than one cell-cycle passes for the tumour cells, so that the cells “see” the fractionated doses as a single, large dose, then the risk of malignancy should be negligible. We estimate the cell division timescale to be on the order of 1–4 days (section “Dose fractionation”). If at least 4 Gray can be delivered each cell cycle, the excess risk of malignancy should be less than the expected lifetime risk, even for pessimistic estimates of *n*_*TSX*_ (see Fig. 8).

Slow-growing schwannomas can probably be treated safely at a minimum dose rate of 1 Gray per day. Faster growing tumours could justify more aggressive treatment: the 3 mm/yr cases from Paldor et al. 2016 might require 4 Gray per day to ensure that we are on the right side of the peak risk from Fig. 8 [6]. It would be interesting to learn how this difference in growth rates is correlated with specific genetic alterations.

This does assume that solid growth can be reliably distinguished from inflammation and “pseudo-progression”, which may not always be possible.

For completeness, we also studied the “worst case scenario” in which fractionated therapy is delivered in independent doses several days apart. This would maximise the chance that tumour cells “recover” from the deterministic effects of cell death, and go on to divide again. This should not be the case in practice [69].

**Fig. 9:**
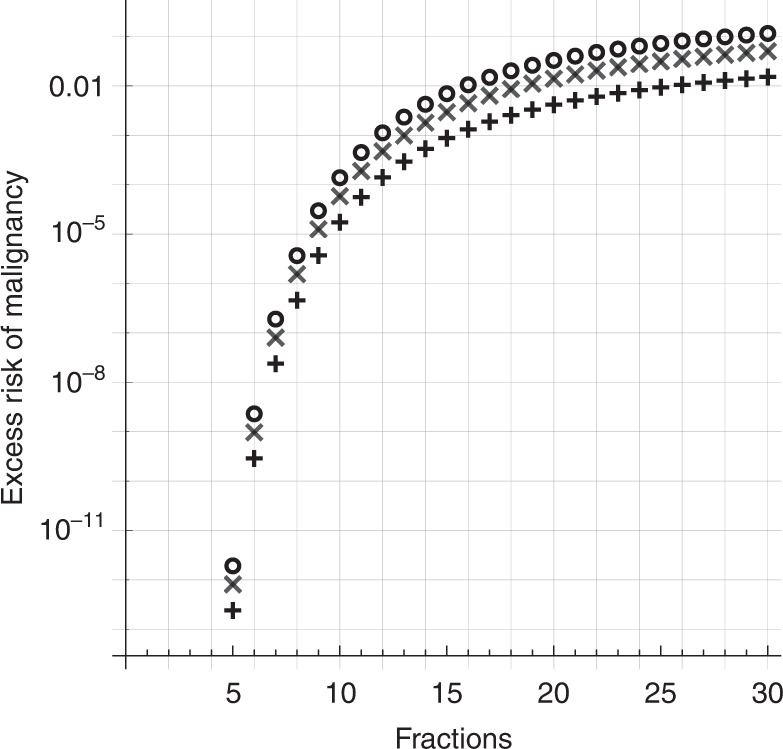
Worst-case scenario excess risk of radiation-associated malignant transformation for tumours of diameters 20mm (+ signs), 30 mm (× signs) and 40 mm (◦ signs) each receiving a dose of 50 Gray, if the two hits are a hypothetical gene with *m*_*TSX*_ = 100. The dose is delivered in separate fractions at least a week apart, maximising tumour cell recovery. There is a very sharp dependence on the number of fractions the dose is split into (*x* axis). This sharp dependence is attributable to the way that the cell survival curve S(D) falls off in Eq. (41).

As the number of fractions increases, the proportion of mutant cells that survive the therapy also increases. As a result, poorly administered hyperfractionated therapy shows a much higher risk for realistic doses and fractions. This risk overtakes the lifetime risk at *F* ≈10 fractions, and may grow as high as ≈10% (see Fig. 9). This enhancement of risk is attributable to the increased survival of mutants following therapy, and not due to enhanced mutagenicity as such (see Fig. 9).

These results must be qualified by the uncertainties in the identity of the hypothetical tumour suppressor (or suppressors) *TSX*, and also in the dynamics of cell division, DNA repair and survival. The assumption that the dose is homogeneous may also be violated for multifocal tumour clusters commonly observed in NF2 patients [20]. In addition, our model for the cell survival curves *S*(*D*) could be improved upon. This may affect our conclusions regarding the “worst-case scenario”, but probably not best practice.

## Discussion

In addition to improved estimates of the underlying mutation rates and dynamics of LOH in Schwann cells, our mechanistic approach to modelling schwannomas has enabled new theoretical estimates of the excess risk of malignant transformation in radiosurgery. This shows that the modelling approach of branching processes on graphs generalises to other neoplasias, enabling new connections and inferences to be made [13]. Furthermore, the uncertainties in this model have raised new questions, creating opportunities for future modelling and experimental work.

Firstly, a better picture of genetic diversity in sporadic VS may be achievable with a more detailed model. This more detailed mechanistic model could include *LZTR1* as one of the first hits, as well as explicitly studying multiple candidate third-hit oncogenes. This could be coupled with a comprehensive study of copy number alterations in VS biopsies, as well as the status of *SMARCB1*, *NF2* and *LZTR1*. This might be achieved by coupling a copy number analysis with an SNP array, or a higher resolution approach leveraging next-generation sequencing [82–85]. A more complex model would require new experiments to determine its parameters, but the method for doing so should be essentially similar to the method in the section “Parameter estimation”: for each gene included in the new model, measure the proportion of cases where pathogenic variant alleles were detected, and compare the frequencies *f*_*gene*_ to those predicted by the model.

One important limitation of our approach is that the mutation rate *u* does not distinguish between transitions and transversions, and the underlying model assumes all substitutions are equally likely [28, 29]. Different base-pair substitutions are known to have different frequencies in different cancers, but data specific to schwannoma does not yet seem to be available [87]. In the absence of sequence-specific data on transition/transversion frequencies in schwannoma, multi-parameter models of substitutions will run into problems of overfitting. However, a recent study of meningioma, a related tumour, suggests that data on transition and transversion frequencies could be derived in a future study of schwannoma with existing technology and methods [88]. This would allow much more detailed inferences about substitution rates than the single parameter *u* reported here.

When applied to malignant transformation, our approach showed that the observed lifetime risk can be explained by a simple two-hit model in the growing tumour (see Fig. 6). This theory also predicts excess LOH on the locus of the tumour suppressor (or suppressors) responsible, *TSX*. Our estimates of *n*_*TSX*_ in section “Parameter estimation” suggest that a promising experimental approach could be to look for LOH in at least ten samples of malignant schwannomas. This might uncover new risk factors, new roles for risk factors known from other tumours such as *TP53*, as well as markers of malignant transformation. The rarity of malignancy in vestibular schwannoma could also provide an opportunity to observe malignant transformation “in slow motion”, with a large and definite reference population of benign tumours.

The extension of this modelling approach to radiotherapy suggested that at tumour sizes and radiation doses typical of therapy, the excess risk of malignancy for sporadic schwannoma is negligible. Cells that receive high doses, and thus have the highest likelihood of induced mutations, will be the least likely to survive. Stochastic effects, which include malignancy, should peak at intermediate doses of around 1 Gray. Above about 4 Gray, deterministic effects dominate, and cell survival falls off very rapidly. This dose is well below recommended prescriptions [70, 89].

The excess risk should also be very small for fractionated therapies, as long as dose rates are higher than 4 Gray over the course of one tumour cell cycle, which we estimate at around 4 days (see section “Dose fractionation”). This is broadly in line with current recommendations. Current clinical practice is therefore expected to have a negligible excess risk of radiation-induced malignancy, consistent with previous empirical studies [3, 9, 86].

The highly pessimistic worst-case scenario in which tumour cells fully recover in between doses should be completely avoidable if the most rapidly growing tumours are treated more aggressively (3mm/year expansion = 4 Gray/day) than more common cases. This pessimistic scenario, detailed in section “Dose fractionation”, is unlikely. Nonetheless, it may be interesting to investigate whether there is an association between malignancy and failure to complete treatment. It should also be cautioned that we do not consider cases of familial NF2.

These findings need to be qualified by the uncertainties both in the identity of the relevant genes. These are questions for which experimental answers are urgently needed. As well as revealing new connections between genomics, epidemiology, and microscopic mechanisms, this work underscores the critical need to identify the genes responsible for both tumour initiation and malignant transformation.

## Supplementary information

counter.py

bootstrap.py

Mathematical and methodological appendices

Reproducibility checklist

Legends to the supplemental material

## Data Availability

The datasets generated and analysed during this study are available from the corresponding author on reasonable request.

## Code availability

All relevant Python code is available in the supplemental material, alongside instructions regarding versioning and dependencies.
