# Supplementary material for: A mechanistic mathematical model of initiation and malignant transformation in sporadic vestibular schwannoma
Source: Br J Cancer. 2022 Sep 12;127(10):1843–57. doi: 10.1038/s41416-022-01955-8 (PMC9643471; doi:10.1038/s41416-022-01955-8)
Supplement: Supplementary file 3 — Mathematical and methodological appendices [file 41416_2022_1955_MOESM3_ESM.pdf]

# Appendices

## A Series solution of sporadic incidence model

Equation (1) can be expressed explicitly:

$$
\begin{aligned}
\frac{dN_{WT}}{dt} &= -\left(\mu_{NF2} + \mu_{SMARCB1} + \mu_{GFX} + r_{LOH}\right) N_{WT} \\
\frac{dN_a}{dt} &= \mu_{GFX} N_{WT} - \left(\mu_{NF2} + r_{LOH}\right) N_a \\
\frac{dN_b}{dt} &= r_{LOH} N_{WT} - \left(\frac{1}{2}\mu_{NF2} + \mu_{GFX} + \frac{1}{2}\mu_{SMARCB1}\right) N_b \\
\frac{dN_c}{dt} &= \mu_{NF2} N_{WT} - \left(\frac{1}{2} r_{LOH} + \mu_{GFX} + \frac{1}{2}\mu_{SMARCB1} + \frac{1}{2}\mu_{NF2}\right) N_c \\
\frac{dN_d}{dt} &= \mu_{SMARCB1} N_{WT} - \left(\frac{1}{2} r_{LOH} + \frac{1}{2}\mu_{NF2}\right) N_d \\
\frac{dN_e}{dt} &= r_{LOH} N_a + \mu_{GFX} N_b - \frac{1}{2}\mu_{NF2} N_e \\
\frac{dN_f}{dt} &= \mu_{NF2} N_a + \mu_{GFX} N_c - \left(\frac{1}{2} r_{LOH} + \frac{1}{2}\mu_{NF2}\right) N_f \\
\frac{dN_g}{dt} &= \frac{1}{2}\mu_{NF2} N_b + \frac{1}{2} r_{LOH} N_c - \left(\frac{1}{2}\mu_{SMARCB1} + \mu_{GFX}\right) N_g \\
\frac{dN_h}{dt} &= \frac{1}{2}\mu_{SMARCB1} N_b + \frac{1}{2} r_{LOH} N_d - \frac{1}{2}\mu_{NF2} N_h \\
\frac{dN_i}{dt} &= \frac{1}{2}\mu_{SMARCB1} N_c + \frac{1}{2}\mu_{NF2} N_d - \frac{1}{2} r_{LOH} N_i \\
\frac{dN_j}{dt} &= \frac{1}{2}\mu_{NF2} N_c - \mu_{GFX} N_j
\end{aligned} \tag{50}
$$

with the matrix $M$ in (1) encoding the dependency structure.

The system of differential equations (1) is linear, and solutions can be found by a wide variety of well-studied methods. Exact symbolic solutions are available but not very useful in our case. The evolutionary dynamics are neutral, with no exponential clonal expansion during initiation. Furthermore, we are interested only in relatively early times $t$ compared to $r_{LOH}^{-1} \approx 300{,}000$

years. Our initial conditions are also not on a singular point [94, 95]. These facts make a power series solution appropriate. This also makes the similarity of the three-hit model to the classic Armitage-Doll curve very clear [4, 23].

Write

$$N_m(t) = \sum_{n=0}^{\infty} A_{m,n} t^n$$

for subpopulation $m$ (see figure 2 and system (1)). The $A_{m,n}$ coefficients can then be found recursively [94].

The resulting solutions, truncated at the third term, read:

$$N_{WT} = N_0 \left(1 - (r_{LOH} + \mu_{GFX} + \mu_{NF2} + \mu_{SMARCB1})t + \mathcal{O}(t^2)\right)$$

$$N_a = N_0 \left(\mu_{GFX} t - \frac{1}{2}\mu_{GFX}(2r_{LOH} + \mu_{GFX} + 2\mu_{NF2} + \mu_{SMARCB1})t^2 + \mathcal{O}(t^3)\right)$$

$$N_b = N_0 \left(r_{LOH} t - \frac{1}{4}(2r_{LOH} + 4\mu_{GFX} + 3\mu_{NF2} + 3\mu_{SMARCB1})r_{LOH} t^2 + \mathcal{O}(t^3)\right)$$

$$N_c = N_0 \left(\mu_{NF2} t - \frac{1}{4}(3r_{LOH} + 4\mu_{GFX} + 3\mu_{NF2} + 3\mu_{SMARCB1})\mu_{NF2} t^2 + \mathcal{O}(t^3)\right)$$

$$N_d = N_0 \left(\mu_{SMARCB1} t - \frac{1}{4}(3r_{LOH} + 2\mu_{GFX} + 3\mu_{NF2} + 2\mu_{SMARCB1})\mu_{SMARCB1} t^2 + \text{[illegible]}\right.$$

$$N_e = N_0 \left(r_{LOH}\mu_{GFX} t^2 - \frac{1}{12}(6r_{LOH} + 6\mu_{GFX} + 9\mu_{NF2} + 5\mu_{SMARCB1})r_{LOH}\mu_{GFX} t^3 \text{[illegible]}\right.$$

$$N_f = N_0 \left(\mu_{GFX}\mu_{NF2} t^2 - \frac{1}{12}(9r_{LOH} + 6\mu_{GFX} + 9\mu_{NF2} + 5\mu_{SMARCB1})\mu_{GFX}\mu_{NF2} t^3 \text{[illegible]}\right.$$

$$N_g = N_0 \left(\frac{1}{2} r_{LOH}\mu_{NF2} t^2 - \frac{1}{24}(5r_{LOH} + 12\mu_{GFX} + 6\mu_{NF2} + 8\mu_{SMARCB1})r_{LOH}\mu_{NF2} t^3 + \mathcal{O}(t^4)\right)$$

$$N_h = N_0 \left(\frac{1}{2} r_{LOH}\mu_{SMARCB1} t^2 - \frac{1}{24}(5r_{LOH} + 6\mu_{GFX} + 8\mu_{NF2} + 5\mu_{SMARCB1})r_{LOH}\mu_{SMARCB1} t^3 + \mathcal{O}(t^4)\right)$$

$$N_i = N_0 \left(\frac{1}{2}\mu_{NF2}\mu_{SMARCB1} t^2\right.$$

$$-\frac{1}{24}(8r_{LOH}+6\mu_{GFX}+6\mu_{NF2}+5\mu_{SMARCB1})\mu_{NF2}\mu_{SMARCB1}t^3+\mathcal{O}(t^4)\Big)$$

$$N_j = N_0\left(\frac{1}{4}\mu_{NF2}^2 t^2 -\frac{1}{8}(r_{LOH}+2\mu_{GFX}+\mu_{NF2}+\mu_{SMARCB1})\mu_{NF2}^2 t^2+\mathcal{O}(t^4)\right) \tag{51}$$

and so, $P_1$, $P_2$ and $P_3$ read

$$\begin{aligned}
P_1 &\approx \frac{1}{2}N_0 r_{LOH}\mu_{GFX}\mu_{NF2}t^3 \\
&- \frac{1}{48}N_0 r_{LOH}\mu_{GFX}\mu_{NF2}(10r_{LOH}+12\mu_{GFX}+12\mu_{NF2}+9\mu_{SMARCB1})t^4+\mathcal{O}(t^5) \\
P_2 &\approx \frac{1}{4}N_0\mu_{GFX}\mu_{NF2}^2 t^3 \\
&- \frac{1}{24}N_0\mu_{GFX}\mu_{NF2}^2(3r_{LOH}+3\mu_{GFX}+3\mu_{NF2}+2\mu_{SMARCB1})t^4+\mathcal{O}(t^5) \\
P_3 &\approx \frac{1}{4}N_0 r_{LOH}\mu_{NF2}\mu_{SMARCB1}t^3 \\
&- \frac{1}{96}N_0 r_{LOH}\mu_{NF2}\mu_{SMARCB1}(9r_{LOH}+12\mu_{GFX}+10\mu_{NF2}+9\mu_{SMARCB1})t^4 \\
&+\mathcal{O}(t^5)
\end{aligned} \tag{52}$$

It is also clear that $f_{LOH}$ and $f_{SMARCB1}$ are only approximately independent of patient age:

$$\begin{aligned}
f_{LOH} &= \frac{P_1+P_3}{P_1+P_2+P_3} \\
&\approx \frac{2\mu_{GFX}r_{LOH}+\mu_{SMARCB1}r_{LOH}}{2\mu_{GFX}r_{LOH}+\mu_{SMARCB1}r_{LOH}+\mu_{GFX}\mu_{NF2}} \\
&\quad +\alpha t+\mathcal{O}(t^2) \\
f_{SMARCB1} &= \frac{P_3}{P_1+P_2+P_3} \\
&\approx \frac{\mu_{SMARCB1}r_{LOH}}{2\mu_{GFX}r_{LOH}+\mu_{SMARCB1}r_{LOH}+\mu_{GFX}\mu_{NF2}} \\
&\quad +\beta t+\mathcal{O}(t^2)
\end{aligned} \tag{53}$$

where

$$\alpha = \frac{r_{LOH}(4\mu_{GFX}+3\mu_{SMARCB1})-\mu_{SMARCB1}(2\mu_{GFX}-2\mu_{NF2}+\mu_{SMARCB1})}{24(2r_{LOH}\mu_{GFX}+\mu_{GFX}\mu_{NF2}+r_{LOH}\mu_{SMARCB1})^2}\mu_{GFX}\mu_{NF2}r_{LOH}$$

$$\beta = \frac{\mu_{SMARCB1}(2r_{LOH}^2\mu_{GFX}+7r_{LOH}\mu_{GFX}\mu_{NF2}+2\mu_{GFX}\mu_{NF2}^2-\mu_{GFX}\mu_{NF2}\mu_{SMARCB1})}{24(2r_{LOH}\mu_{GFX}+\mu_{GFX}\mu_{NF2}+r_{LOH}\mu_{SMARCB1})^2}r_{LOH}$$

It follows that this approximation is accurate up to a factor of approximately $\mathcal{O}(r_{LOH}t)$, so any age-dependence should only show up on a timescale of $r_{LOH}^{-1} \approx 300,000$ years. This is far in excess of a typical human lifetime, so in any real empirical study, any association can be expected to be unobservable.

This assumes that these alterations are selectively neutral. If this is not true, then the relative frequency of these alterations may change over time, indicating selection [81]. It may also be the case that the model ceases to be accurate over shorter timescales than $r_{LOH}^{-1}$: without knowledge of selection coefficients of the relevant genes, we cannot comment further.

# B Statistical methods: bootstrapping and regularisation

Bootstrapping consists of drawing new samples at random from a dataset, with replacement [47, 48]. Our measures of relative frequency come from datasets with $n$ samples, $k$ of which are positive results. When one sample is drawn uniformly at random, there is a probability $p$ that this draw will be positive.

Bootstrapping $n$ samples with this probability $p$ will result in a "sample" value $\hat{k}$. Because this sampling process consists of $n$ independent draws with replacement, the resulting sample value $\hat{k}$ will be binomially distributed:

$$\Pr(\hat{k}) = \binom{n}{\hat{k}} p^{\hat{k}} (1-p)^{\hat{k}}$$

These draws can therefore be simulated by generating binomially distributed random numbers, given an estimate of the parameter $p$.

Binomial random variables were generated with Python's NumPy library (https://numpy.org/about/). For each sample value $\hat{k}$, a sample frequency estimator $\hat{f}$ can be calculated:

$$\hat{f} = \frac{\hat{k}}{n} \tag{54}$$

and substituted for $f_{LOH}$ or $f_{SMARCB1}$ in formulae (13) and (14). Repeating this process for many values of $\hat{f}_{LOH}$ and $\hat{f}_{SMARCB1}$ allowed us (in theory) to generate distributions for $n_{GFX}$ and $r_{LOH}$ that reflect the uncertainty due to the small sample size of the underlying experiment.

However, a problem arises if $\hat{k}$ is 0 or $n$: when $\hat{f}_{SMARCB1} = 0$ or $\hat{f}_{LOH} = 1$, equations (13) and (14) are singular. This can make distribution means, variances, and even some quantiles ill-defined. To address this, we used a Bayesian estimator for $\hat{f}$ given $\hat{k}$ and $n$ [42, 96],

$$\hat{f} = \frac{\hat{k} + \alpha}{n + \alpha + \beta} \tag{55}$$

for some $\alpha = \beta > 0$. The exact choice of constant $\alpha = \beta$ was not found to have a strong effect on our estimates or computed confidence intervals: we chose the Jeffreys prior, $\alpha = \beta = \frac{1}{2}$, so that

$$\hat{f}_{\text{Jeffreys}} = \frac{\hat{k} + \frac{1}{2}}{n+1}$$

This gives a rigorous justification for additive smoothing: it ensures that our prior distribution is uninformative, and regularises the sample estimator $\hat{f}$, making it well-behaved when $\hat{k}$ is 0 or $n$ [42]. Intuitively, this can be thought of as adding one additional datum with a value of $\frac{1}{2}$ to the resampled dataset.

It should be noted that we already apply additive smoothing with $\alpha = \frac{1}{2}$ to our estimate of the parameter $p$ in 2.1.4 before the bootstrapping procedure. This avoids the case in which $p = 0$, in which case the results would be meaningless: it is impossible to meaningfully resample data with no events. This is therefore not a true bootstrapping procedure, in which we resample the underlying data. It is instead a simulation of a bootstrapping procedure applied to a regularised dataset. Namely, this regularised dataset is equivalent to adding one extra datum to the original, with a value of $\frac{1}{2}$.

It is easily seen that this procedure becomes equivalent to bootstrapping in the limit that $\alpha = 0$.

We also considered and rejected the use of an informative prior. Because the regularisation procedure is applied both before and after resampling, an uninformative prior should be used in both cases, so as not to introduce inappropriate bias. From a Bayesian perspective, while there are good reasons to believe that $f_{LOH}$ and $f_{SMARCB1}$ are neither exactly zero nor exactly one, there is no objective reason to pick a particular value otherwise.

The choice of prior is easily adjusted to see what effect it has on our conclusions: our computed confidence intervals are relatively robust. The Python code that implements the bootstrapping procedure has been included in the supplemental material.

# C Parameter sensitivity

The parameters of the model and experimental results (consisting of the epidemiological data of Evans et al. 2005, LOH(22q) data M. Carlson et al. 2018 and the present work's results on *SMARCB1*) can be gathered into subsets consisting of the parameters that can be determined or measured directly, or "inputs", $X = \{A, N_0, b, f_{LOH}, f_{SMARCB1}\}$; and the parameters that we predict theoretically, the "outputs" $Y = \{n_{GFX}, r_{LOH}, u\}$. Estimating the sensitivity of parameters for non-linear models where the variables have no explicit form is an open research topic [97, 98]. In our case, our model is a system of linear ordinary differential equations, and explicit forms are available for all parameter dependencies. As such, the change in theoretical outputs with respect to changes in the inputs is straightforwardly represented by the Jacobian $J$ of the outputs with respect to the inputs [46].

Using the explicit expression for $u$

$$u = \frac{1}{b}\left(\frac{8A}{N_0 n_{NF2}^2 n_{SMARCB1}}\left(\frac{f_{SMARCB1}(1-f_{LOH})}{(f_{LOH}-f_{SMARCB1})(1+f_{LOH})}\right)\right)^{1/3}$$

the Jacobian $J$ can be computed:

$$\begin{pmatrix} 0 & 0 & 0 & \frac{n_{SMARCB1}}{2f_{SMARCB1}} & -\frac{f_{LOH}n_{SMARCB1}}{2f_{SMARCB1}^2} \\ \frac{n_{NF2}(f_{LOH}-f_{SMARCB1})bu}{6A(1-f_{LOH})} & -\frac{n_{NF2}(f_{LOH}-f_{SMARCB1})bu}{6(1-f_{LOH})N_0} & 0 & \frac{n_{NF2}\left(f_{LOH}^2-3f_{LOH}f_{SMARCB1}+f_{LOH}-f_{SMARCB1}+2\right)bu}{6(f_{LOH}-1)^2(f_{LOH}+1)} & -\frac{n_{NF2}(f_{LOH}-3f_{SMARCB1})C}{3(f_{LOH}-1)f_{SMARCB1}} \\ \frac{u}{3A} & -\frac{u}{3N_0} & -\frac{u}{b} & -\frac{\left(f_{LOH}^2-2f_{LOH}+2f_{SMARCB1}-1\right)u}{3(f_{LOH}-1)(f_{LOH}+1)(f_{LOH}-f_{SMARCB1})} & \frac{f_{LOH}u}{3f_{SMARCB1}(f_{LOH}-f_{SMARCB1})} \end{pmatrix}$$

where $C = \sqrt[3]{-\frac{A(f_{LOH}-1)f_{SMARCB1}}{(f_{LOH}+1)N_0 n_{NF2}^2 n_{SMARCB1}(f_{LOH}-f_{SMARCB1})}}$. Substituting numerical values yields

$$J = \begin{pmatrix} 0 & 0 & 0 & 2833.33 & -137889. \\ 24856.1 & -1.23 \times 10^{-12} & 0 & 5.40 \times 10^{-6} & 0.00 \\ 5.45 & -2.70 \times 10^{-16} & -1.44 \times 10^{-11} & -7.00 \times 10^{-10} & 8.38 \times 10^{-9} \end{pmatrix} \tag{57}$$

Many values in the analytical Jacobian are exactly zero. This warrants several comments. It is remarkable that $n_{GFX}$ is completely insensitive to estimates of $A$, $N_0$ or $b$. This implies that improved measurements of epidemiological data, imaging, or precursor cell dynamics are likely to have no effect on the predicted properties of the underlying hypothetical gene *GFX*.

It is also remarkable that $r_{LOH}$ is completely insensitive to changes in the value of $b$. This suggests that the dynamics of LOH are better constrained by epidemiological data than microscopic estimates, as $r_{LOH}$ depends on $A$ but not on $b$.

Clearly, all outputs appear to be most sensitive to changes in $f_{LOH}$ and $f_{SMARCB1}$, which strongly reinforces our recommendation to increase the precision with which these parameters can be measured. Namely, by a follow-up experiment with a larger sample size.
