# Supplementary material for: A mechanistic mathematical model of initiation and malignant transformation in sporadic vestibular schwannoma
Source: Br J Cancer. 2022 Sep 12;127(10):1843–57. doi: 10.1038/s41416-022-01955-8 (PMC9643471; doi:10.1038/s41416-022-01955-8)
Supplement: Supplementary file 5 — Legends to the supplemental material [file 41416_2022_1955_MOESM5_ESM.docx]

Supplementary Material legends

1. counter.py: Python script that counts the number of sites sensitive to truncation and nonsense mutations from an EMBL file.
2. bootstrap.py: Python script to bootstrap posterior distributions for model parameter estimates, return confidence intervals, and plot the distributions.
3. Appendices.pdf: Mathematical and methodological appendices, detailing the form and solution of equation (1), limits of validity of the series expansion, and statistical methodology (bootstrapping and additive smoothing).
4. Legends.docx: this file, the legends to the Supplemental Material.
